# Accuracy of diagnostic strategies for detecting *Schistosoma mansoni* infection in Brazil: A systematic review and meta-analysis

**DOI:** 10.1590/0037-8682-0466-2025

**Published:** 2026-08-03

**Authors:** Tália Santana Machado de Assis, Mariana Lourenço Freire, Sarah Nascimento Silva, Daniel Moreira de Avelar, Gláucia Cota, Ana Rabello

**Affiliations:** 1 Fundação Oswaldo Cruz, Instituto René Rachou, Pesquisa Clínica e Políticas Públicas em Doenças Infecto-Parasitárias, Núcleo de Avaliação de Tecnologias em Saúde, Belo Horizonte, MG, Brasil.; 2 Centro Federal de Educação Tecnológica de Minas Gerais, Contagem, MG, Brasil.

**Keywords:** Diagnostic test, Schistosoma mansoni, Systematic review

## Abstract

**Background:**

*Schistosoma mansoni* infection remains a major public health concern in Brazil. Accurate diagnostic methods are essential for monitoring transmission and guiding control and elimination strategies. This systematic review and meta-analysis aimed to assess the performance of diagnostic tests used for *S. mansoni* infection in Brazil.

**Methods:**

Studies conducted in endemic areas of Brazil that used parasitological methods as the reference standard, reported sensitivity and specificity, and were published through October 2024, were included in the analysis. Diagnostic performance was synthesized using univariate and multivariate meta-analyses, and risk of bias was assessed with QUADAS-2.

**Results:**

Thirty-three studies were included; most were conducted in Minas Gerais (63%). The main reference standard was Kato-Katz alone (48%) or combined with other parasitological methods (42.4%). Enzyme-linked immunosorbent (ELISA)-SEA results showed higher accuracy than ELISA-SWAP (81% vs 68%), although both exhibited heterogeneity above 80%. Point-of-care circulating cathodic antigen with trace results interpreted as positive (POC-CCA^+^) or negative (POC-CCA^-^) showed similar accuracy (70% vs 73%, respectively), with heterogeneity exceeding 80%. For conventional PCR, PCR-ELISA, real-time PCR, and loop-mediated isothermal amplification (LAMP), accuracy ranged from 67% to 82% (heterogeneity: > 90%. In multivariate analysis, conventional PCR had the highest diagnostic odds ratio (DOR: 99.4), followed by PCR-ELISA (DOR: 12.4), whereas the two POC-CCA reading criteria resulted in lower values (trace-positive: 8.5, trace-negative: 6.1).

**Conclusions:**

Overall, these observations support the use of combined diagnostic strategies and underscore the need for multicenter, methodologically rigorous studies to strengthen surveillance, control, and elimination efforts for *Schistosoma mansoni* **i**nfection in Brazil.

## INTRODUCTION

Infection with the trematode *Schistosoma mansoni* causes a major parasitic disease of global public health concern and remains widely distributed across tropical and subtropical regions^1^ ^-^ ^4^. Although schistosomiasis-related morbidity and mortality have decreased over the past decades, the disease remains endemic in several parts of Brazil, particularly in the Northeast and Southeast, where it disproportionately affects socioeconomically vulnerable populations^1^ ^,^ ^5^ ^-^ ^7^. Transmission occurs through skin contact with freshwater contaminated by cercariae released from infected snails. Most infected individuals develop the asymptomatic intestinal form, whereas severe disease is characterized by periportal fibrosis, leading to hepatosplenomegaly and gastrointestinal varices^8^.

Early and accurate diagnosis is essential for patient management, interrupting transmission, and evaluating control and elimination strategies^1^ ^,^ ^9^ ^,^ ^10^. However, currently available tests have important limitations in accuracy and accessibility, particularly for individuals with low parasite burdens^11^. Mild, chronic, and asymptomatic infections are often undetected by existing diagnostic tools^3^, underscoring the need for more sensitive diagnostic methods to reliably assess disease burden, treatment response, and transmission dynamics. This need is especially critical in settings implementing mass drug administration under the World Health Organization (WHO) neglected tropical diseases roadmap to eliminate schistosomiasis as a public health problem by 2030^12^.

Given the ongoing challenges in accurate diagnosis, WHO guidelines for the control and elimination of human schistosomiasis recommend complementary strategies to improve sensitivity in low-endemicity settings, including examination of multiple parasitological slides and the use of immunological or molecular tests when available^3^. 

The Kato-Katz method has traditionally been recommended because it is simple, inexpensive, and operationally feasible in field settings^13^ ^,^ ^14^. However, given that its performance depends on parasite burden, its sensitivity is limited in low-prevalence settings^15^ ^,^ ^16^, which may lead to underdiagnosis and compromise disease control efforts^17^. To address this limitation, alternative and complementary approaches have been proposed, including immunological assays, such as enzyme-linked immunosorbent assay (ELISA)^15^ ^,^ ^18^ ^,^ ^19^ and rapid immunochromatographic tests (ICTs) for detecting circulating antigens, including the point-of-care circulating cathodic antigen (POC-CCA)^9^ ^,^ ^20^. Molecular methods, particularly conventional and real-time PCR^21^ ^-^ ^24^ and loop-mediated isothermal amplification (LAMP)^25^ ^-^ ^27^, have also shown potential for improving diagnostic accuracy, particularly in low-transmission settings. Diagnostic approaches that combine parasitological, immunological, and molecular methods may improve overall accuracy and reduce false-negative results^28^ ^-^ ^30^.

Despite the growing number of studies evaluating new diagnostic methods, substantial heterogeneity in study populations, methodological designs, and reference standards continues to hamper direct comparisons and the development of consistent recommendations for large-scale implementation^16^ ^,^ ^17^ ^,^ ^31^ ^,^ ^32^. A comparative synthesis of the available evidence is therefore needed to inform epidemiological surveillance and support the elimination of schistosomiasis as a public health problem in Brazil. This is particularly relevant in a country marked by heterogeneous transmission patterns and ongoing control efforts, which complicate both accurate diagnosis and the interpretation of test performance. Accordingly, this systematic review and meta-analysis evaluate diagnostic accuracy studies of *S. mansoni* infection conducted in Brazil.

## METHODS

### Protocol and registration

This systematic review was conducted and reported in accordance with the *Cochrane Handbook for Systematic Reviews of Diagnostic Test Accuracy* ^33^ and the Preferred Reporting Items for Systematic Reviews and Meta-analyses (PRISMA) guidelines^34^. The research question was formulated using the PICO (population, intervention, comparator, and outcome) framework. This review is registered in the Open Science Framework (OSF) Registries (https://osf.io/mejv5). 

### Eligibility criteria

The review was guided by the following question: “*What is the performance of diagnostic tests for S. mansoni infection in Brazil*?” Based on the PICO framework, the inclusion criteria were: population (P), patients at risk of *S. mansoni* infection; intervention (I), any diagnostic test for *S. mansoni* infection, including ELISA and indirect immunofluorescence assay (IFA), rapid tests such as POC-CCA, circulating anodic antigen (CAA) detection, parasitological examination, PCR, PCR-ELISA, and LAMP; comparator (C), any parasitological test, regardless of the number of slides analyzed or samples tested; and outcome (O), diagnostic accuracy measures, including sensitivity, specificity, and related performance indicators.

Original studies conducted in Brazil that reported the performance of diagnostic strategies for patients suspected of *S. mansoni* infection were included. Studies were excluded if they: (a) included fewer than 10 schistosomiasis cases, increasing the risk of small-sample bias; (b) lacked essential information on the index test or reference standard; (c) assessed populations from other countries without stratified data for Brazil; (d) used the same tests as both the index test and the reference standard; (e) did not report both sensitivity and specificity; or (f) included a control group composed of individuals not residing in endemic areas. 

### Information sources and search strategies

MEDLINE (via PubMed), EMBASE, and the Virtual Health Library (VHL) were searched for records published through October 1, 2024, with no language or date restrictions. Search strategies were tailored to each database using controlled descriptors (MeSH, Emtree, DeCS) and free-text terms covering four components: population (*S. mansoni* infection), intervention (diagnostic tests), outcome (sensitivity, specificity, and other measures of diagnostic performance), and location (Brazil), combined with Boolean operators (OR, AND). For the outcome component, a validated filter for diagnostic test accuracy studies, as recommended by the National Library of Medicine (NLM), was applied^35^. Full search strategies are provided in Supplementary File 1 (S1 File).

### Study selection process

Records were first exported to Mendeley Reference Management Software^36^ for duplicate removal and then imported into Rayyan^37^ where duplicates were checked again and titles and abstracts were screened. Two reviewers (MLF, TSMA) independently performed this step using based on the predefined inclusion and exclusion criteria. Discrepancies were resolved by consensus or, when necessary, with input from two additional reviewers (GC, ALTR). Full-text articles were then assessed for eligibility and data extraction.

### Data extraction

Data were extracted independently by two researchers (MLF, TSMA). The following information was collected: author, year, and study location; study design and sample size; population characteristics; type and specifications of the index test; reference standard; and raw test data, including true positives (TPs), false positives (FPs), false negatives (FNs), and true negatives (TNs). All data were recorded in pre-specified Microsoft Excel spreadsheets.

### Data analysis and synthesis of results

The primary measures of diagnostic performance were sensitivity and specificity, extracted directly from the studies or calculated from 2 × 2 contingency tables. Since these measures are intrinsically interdependent, diagnostic performance was conceptualized and analyzed based on their combined interpretation rather than as isolated parameters. Diagnostic accuracy, defined as the proportion of correctly classified cases, was reported as a complementary measure but was not used as the primary basis for comparison, as it depends on disease prevalence.

Given the methodological diversity and the varying number of studies available for each diagnostic test, both univariate and multivariate (bivariate) meta-analytic approaches were applied. 

Whenever sufficient studies were available, joint modeling was prioritized to provide a more robust and clinically meaningful synthesis of diagnostic performance. For comparisons with at least four studies, multivariate (bivariate) random-effects models were applied^38^. This approach simultaneously estimates sensitivity and specificity while accounting for their correlation and between-study heterogeneity. Models were implemented using a generalized linear mixed-effects model (GLMM) on the logit scale, from which summary estimates of sensitivity, specificity, diagnostic odds ratio (DOR), summary receiver operating characteristic (SROC) curves, and derived accuracy were obtained.

For comparisons with fewer than four studies, joint bivariate modeling was considered unreliable. In these cases, sensitivity and specificity were synthesized using univariate random-effects models or presented descriptively, while preserving their paired interpretation. Although this approach does not explicitly model the correlation between sensitivity and specificity, it allowed inclusion of all eligible evidence. Statistical heterogeneity was assessed with the I² statistic, and results are reported with 95% confidence intervals (CIs). Threshold effects were not modeled explicitly due to inconsistent reporting of positivity criteria across studies.

Meta-analyses were conducted using Meta-DiSc version 2.0^39^ which implements a bivariate GLMM via the *glmer* function in the *lme4* package^40^ and Meta-Analysis Online^41^. Each population was included only once to avoid data overlap. When multiple accuracy estimates were reported for the same method in the same population, selection followed a predefined hierarchy: (i) the same type most relevant to the intended clinical use and (ii) the platform or protocol most comparable to those used in other included studies. Only when these criteria yielded more than one equally eligible estimate (same methodology, sample, threshold, and primary analysis) was the highest-performing estimate selected as the residual criterion. This approach was intended to improve comparability, avoid double-counting and statistical dependence, and preserve the intrinsic performance of each technology under equivalent conditions.

### Methodological quality assessment

Risk of bias was assessed with the Quality Assessment of Diagnostic Accuracy Studies-2 (QUADAS-2) tool^42^. Two reviewers (MLF, TSMA) performed the assessment independently. QUADAS-2 evaluates four main domains of risk of bias: patient selection, index test, reference standard, and flow and timing. The first three domains were also used to assess applicability to the review question. Signaling questions guided judgments across all domains.

## RESULTS

### Studies included

**FIGURE 1:**
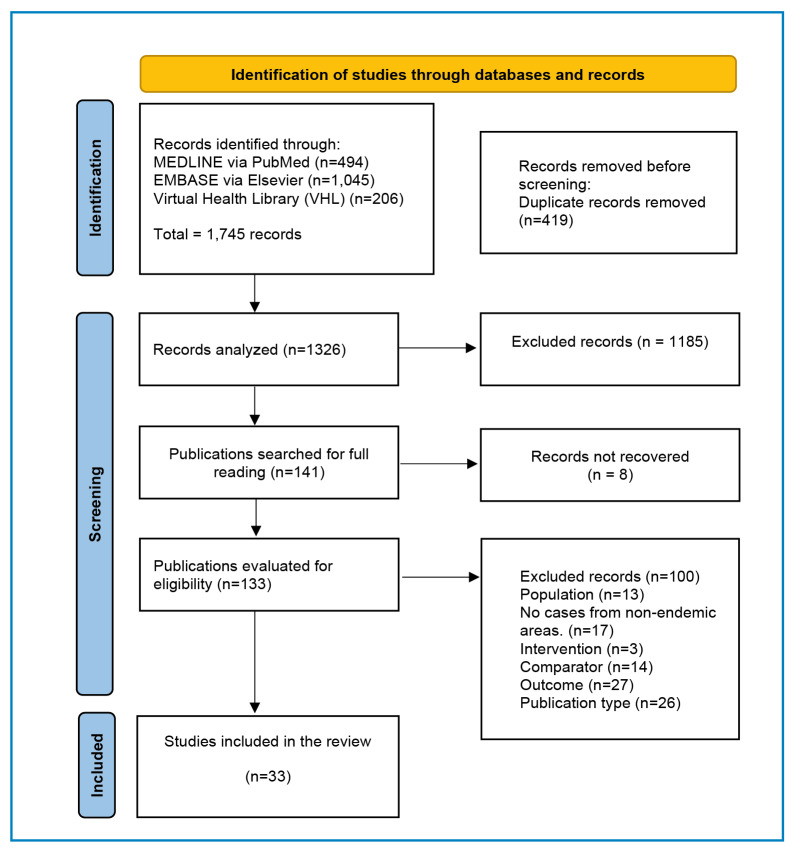
PRISMA flow diagram of the study selection process.

A total of 1,745 records were identified from MEDLINE via PubMed (n = 494), EMBASE via Elsevier (n = 1,045), and VHL (n = 206). After removing 419 duplicates, 1,326 records remained for screening (Figure 1). Of these, 1,185 were excluded after title and abstract review, leaving 141 publications for full-text assessment. Eight articles could not be retrieved, resulting in 133 studies evaluated in full. After full-text review, 100 studies were excluded for the following reasons: inadequate population, including studies with fewer than 10 patients or conducted outside Brazil (n = 13); specificity assessed in individuals from non-endemic areas (n = 17); index test limitations, such as insufficient test description or use of the same test as a comparator (n = 3); non-parasitological comparator (n = 14); absence of sensitivity and specificity outcomes (n = 27); and ineligible publication type, such as systematic reviews or case reports (n = 26). Ultimately, 33 studies met the eligibility criteria and were included in this review. 

### Descriptive analysis of included studies

Molecular assays were the most frequently evaluated tests. Conventional PCR was reported in five studies, followed by PCR-ELISA (three studies), qPCR/real-time PCR (two studies), and LAMP (two studies, including one LAMP-Smits1 variation). Feces were the main specimen used for DNA extraction, except in one study that used urine^43^. Among the 11 studies using fecal samples, 500 mg was the most common amount for DNA extraction (63.6%). Studies employing LAMP extracted DNA from 1-10 g of feces. The QIAamp DNA Stool Mini Kit (Qiagen, Germany) was used in 58.3% of studies. Among serological assays, ELISA was evaluated with different antigens, including in-house ELISA SWAP IgG (four studies), ELISA SEA anti-IgG (three studies), ELISA IgG anti-SmME (one study), and combinations such as ELISA IgG, ELISA IgM, and ICT IgG/IgM (LDBio Diagnostics, France) (one study). Urine antigen detection focused predominantly on POC-CCA, reported in 11 studies using standard or differentiated reading formats (T+ or T−). The TF-Test, a parasitological assay, was described in two studies (Table 1).

**TABLE 1:** Main characteristics of the studies included in the systematic review of diagnostic tests for schistosomiasis in Brazil.

Reference	Patient origin	Reference test(s)	Index test(s)	Sample size(case/non-case)	Age	Male/Female
Pontes et al.^44^	Comercinho, MG	Kato-Katz	PCR	60/134	Mean: 36 years	95/99
Gonçalves et al.^45^	Paracambí, RJ	Kato-Katz or spontaneous sedimentation	ELISA SWAP IgG	16/253	NR	NR
Gomes et al.^46^	Governador Valadares, MG	Kato-Katz	PCR	25/262	Range: 6-89 years	33/34
Gomes et al.^47^	Montes Claros, MG	Kato-Katz	PCR-ELISA	38/168	Range: 1-86 years	99/107
Oliveira et al.^29^	Sumidouro, RJ	Kato-Katz	PCR	34/46	NR	56/46
Frota et al.^28^	Pacoti, CE	Kato-Katz	ELISA SEA	25/262	33 years	132/155
Siqueira et al.^23^	Montes Claros, MG	Kato-Katz	PCR-ELISA	29/172	NR	NR
Siqueira et al.^48^	Montes Claros, MG	Kato-Katz	TF-Test	37/164	NR	NR
Carneiro et al.^15^	Maranguape, CE	Kato-Katz	ELISA SWAP IgG	40/210	Mean: 26 years	150/100
Carvalho et al.^21^	Piau and Coronel Pacheco, MG	Kato-Katz	PCR	16/203	Mean: 38 years	112/107
Enk et al.^43^	Montes Claros, MG	Kato-Katz, saline gradient and miracidia hatching technique	PCR	69/125	Range: 1-88 years	102/92
Siqueira et al.^23^	Montes Claros, MG	Kato-Katz and TF-Test	PCR-ELISA	29/172	Range: 1-96 years	108/93
Coelho et al.^49^	Estreito de Miralta, MG	Kato-Katz and saline gradient	POC-CCA T+	18/66	Range: 1-86 years	38/46
Siqueira et al.^50^	Montes Claros, MG	Kato-Katz or saline gradient	POC-CCA T+	34/107	Range: 1-86 years	67/74
Ferreira et al.^51^	Pains, MG	Kato-Katz	POC-CCA T+	26/274	Mean: 44 years	11/189
Lindholz et al.^16^	Estância, SE	Kato-Katz	POC-CCA T+	55/406	NR	NR
Gandasegui et al. ^25^	Umbuzeiro, PB	Kato-Katz	LAMP	13/149	NR	NR
Oliveira et al.^17^	Januária, MG	Kato-Katz, saline gradient and Helmintex	POC-CCA T−, POC-CCA T+	112/116	Mean: 34.9 years; Median: 32 years	112/135
Nacife et al.^52^	Água Boa and Nacife, MG	Kato-Katz	TF-Test	249/296	Range: 0-86 years	296/249
Queiroz et al.^53^	Montes Claros, MG	Kato-Katz and saline gradient	POC-CCA	112/116	Mean: 34.9 years	122/135
Senra et al.^54^	Montes Claros, MG	Kato-Katz	PCR-ELISA	114/92	Range: 1-86 years	107/99
Grenfell et al.^9^	Estreito de Miralta and Samambaia, MG	Kato-Katz and saline gradient	POC-CCA T−, POC-CCA T+	21/27	Range: 4-81 years	27/21
Souza et al.^55^	Primavera, PA	Kato-Katz	POC-CCA T−, POC-CCA T+	35/338	Range: 7-70 years	186/186
Magalhães et al.^22^	Januária, MG	Kato-Katz or HPJ or saline gradient or Helmintex	qPCR	114/92	Range: 2-88 years	102/113
Siqueira et al.^56^	Montes Claros, MG	Kato-Katz or saline gradient or Kato-Katz + saline gradient	Real-time PCR	15/127 (Estreito de Miralta) and 23/125 (Tábuas)	Range: 1-86 years	140/150
Souza et al.^57^	Turiaçú, MA	Kato-Katz or saline gradient or Kato-Katz + saline gradient	POC-CCA T−	63/154	NR	106/111
Bezerra et al.^20^	Maruim, SE	Kato-Katz	POC-CCA T−, POC-CCA T+	62/65	Mean: 27 years	55/72
Oeyyemi et al.^58^	Montes Claros and Belo Horizonte	Kato-Katz and/or saline gradient	ELISA SWAP IgG, ELISA SEA	30/30	Range: 1-79 years	NR
Gomes et al.^26^	Porto de Galinhas, PE	Kato-Katz	LAMP-SMITS1	144/178	NR	157/165
Magalhães et al.^59^	Januária, MG	Kato-Katz or HPJ or saline gradient or Helmintex	ELISA SWAP IgG, ELISA SEA	118/139	Mean: 32 years	NR
Lopes et al.^60^	Januária and Jaboticatubas, MG; Primavera, PA	Kato-Katz	Anti-SmME IgG ELISA	53/65	Range: 6-69 years	55/75
Pieri et al.^61^	Cametá, PA; Itaquara and Conde, BA; Montes Claros, Malacacheta and Comercinho, MG; Maranguape, CE; Indiaroba, SE	Kato-Katz or HPJ or saline gradient or Helmintex®	POC-CCA T−, POC-CCA T+	424/1471	> 2 years	NR
Ramos et al.^19^	Estância, SE	Helmintex	ELISA IgG, ELISA IgM and ICT IgG/IgM	85/130 (ELISA IgG), 63/137 (ELISA IgM), 70/117 (ICT IgG/IgM)	NR	NR

**Abbreviations: MG:** Minas Gerais; **RJ:** Rio de Janeiro; **CE:** Ceará; **SE:** Sergipe; **PB:** Paraíba; **PA:** Pará; **MA:** Maranhão; **PE:** Pernambuco; **BA:** Bahia.

Case distribution was geographically heterogeneous, with most studies conducted in the Southeast (20/33; 61%), particularly in Minas Gerais (17/33; 51%), followed by the Northeast (9/33; 27.3%). Regarding the reference standard, 19 studies (57.6%) used a single parasitological test, most commonly Kato-Katz (16/33; 48%). The remaining 14 studies (42.4%) used combinations of parasitological methods, all including Kato-Katz (Table 1).

Sample sizes varied widely, from 27 participants to population-based surveys with more than 1,471 individuals. In several studies, the case group was smaller than the control group^21^ ^,^ ^28^. Age and sex were reported inconsistently, but participants ranged from 0 to 96 years and were generally balanced by sex (Table 1).

### Univariate analyses

**FIGURE 2:**
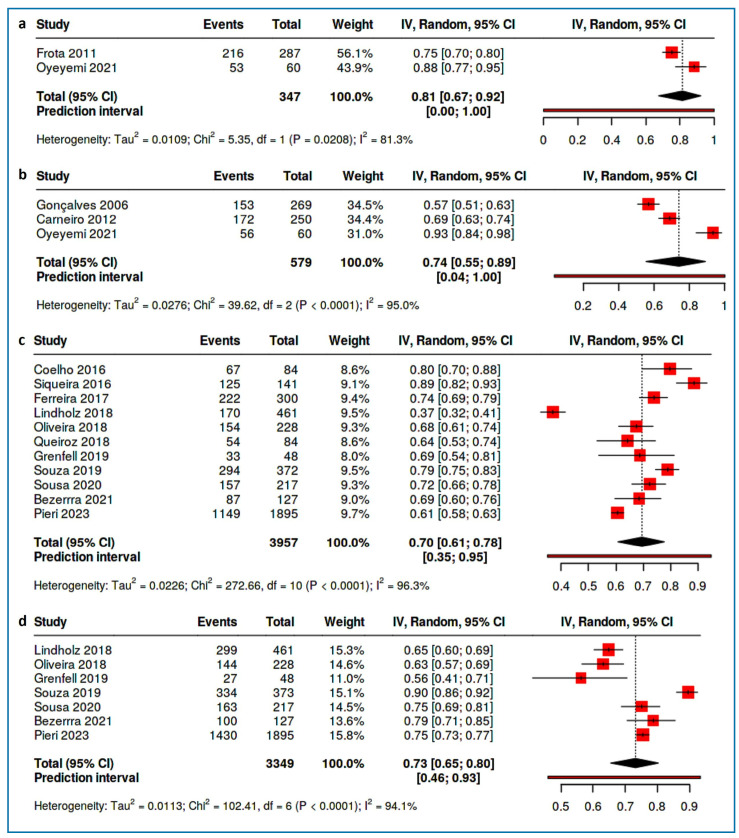
Pooled accuracy estimates of ELISA-SEA **(a)**, ELISA-SWAP **(b)**, POC-CCA with trace results considered as positive **(c)** or negative **(d)** for diagnosing *Schistosoma mansoni* infection in Brazil.

Seven studies evaluated ELISA for diagnosing *S. mansoni* infection using different antigen sources: recombinant multi-epitope antigen^60^, crude adult worm extract^15^ ^,^ ^45^ ^,^ ^58^ ^-^ ^59^, egg extract^28^ ^,^ ^30^ ^,^ ^59^, and a commercial kit (Supplementary File 2 [S2 File])^19^. ELISA-SEA showed higher accuracy (0.81; 95% CI: 0.67-0.92) than ELISA-SWAP (0.74 (0.55-0.89), although both exhibited high heterogeneity (I² > 80%; Figure 2a,b). The study by Magalhães et al.^59^ was excluded from the meta-analysis because it evaluated IgG subclasses (IgG1 and IgG4), whereas other studies assessed total IgG. Only one study of IFA met the eligibility criteria^45^. 

Eleven studies evaluated POC-CCA in urine for diagnosing *S. mansoni* infection in Brazil^9^ ^,^ ^16^ ^,^ ^17^ ^,^ ^20^ ^,^ ^49^ ^,^ ^50^ ^,^ ^51^ ^,^ ^53^ ^,^ ^55^ ^,^ ^57^ ^,^ ^61^. These studies differed in how “trace” results were interpreted, either as positive or negative (Supplementary File 3 [S3 File]). Accuracy was similar under both approaches, although heterogeneity was high in both cases (Figure 2c,d). Another study evaluated a rapid test on serum samples^19^. 

Twelve studies evaluated molecular methods for the diagnosis of *S. mansoni* infection, including conventional PCR^21^ ^,^ ^29^ ^,^ ^43^ ^,^ ^44^, real-time PCR^22^ ^,^ ^23^, and PCR-ELISA (Supplementary File 4 [S4 File])^47^ ^,^ ^48^ ^,^ ^54^. Additionally, two studies assessed LAMP performance^25^ ^,^ ^26^. 

**FIGURE 3:**
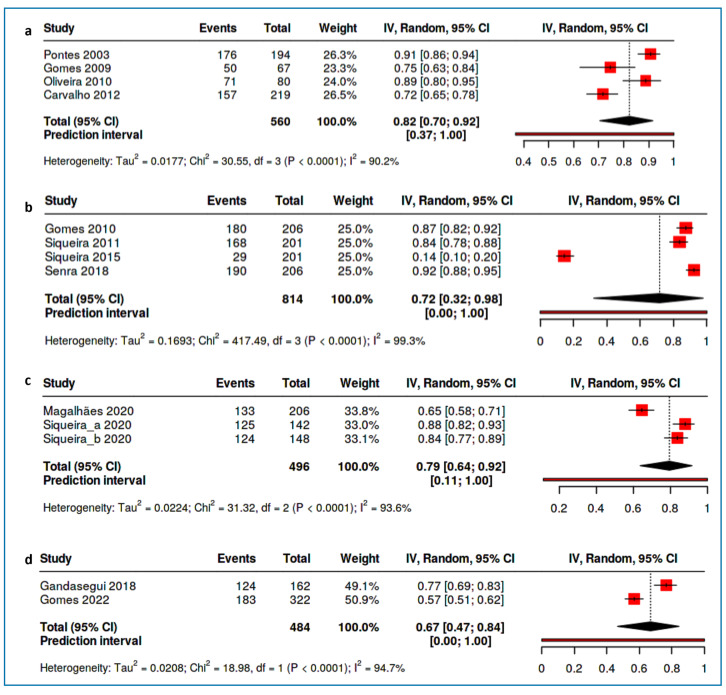
Pooled accuracy estimates of conventional PCR **(a)**, PCR-ELISA **(b)**, real-time PCR **(c)**, and LAMP **(d)** for diagnosing *Schistosoma mansoni* infection in Brazil.

Performance was similar across molecular methods, with accuracy ranging from 67% (95% CI: 47-84%) to 82% (95% CI: 70-92%); however, high heterogeneity was consistently observed (Figure 3). The study by Enk et al.^43^ was excluded from the pooled analysis because, unlike other conventional PCR studies that used stool samples, it used urine samples.

Regarding parasitological tests, studies evaluating Helmintex^16^ and TF-Test^48^ ^,^ ^52^, using Kato-Katz as the reference standard (Supplementary File 5 [S5 File]). In the meta-analysis, TF-Test showed a pooled accuracy of 75% (95% CI: 72-78) across two studies, with no heterogeneity (I² = 0%). One study assessed Kato-Katz using Helmintex as the reference standard^61^. 

Summary analyses of sensitivity and specificity for all tests included in this review are available in Supplementary File 6 (S6 File).

### Multivariate analyses

**FIGURE 4:**
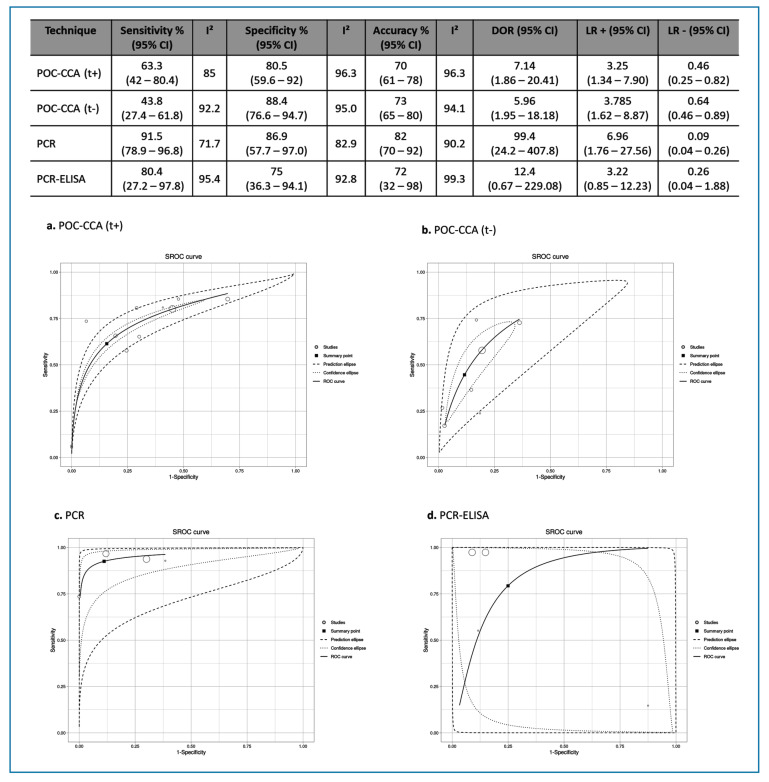
Pooled estimates of sensitivity and specificity from the multivariate analysis of tests evaluated for the diagnosis of schistosomiasis in Brazil. **(a)** SROC curve for POC-CCA with trace results considered positive (T+); **(b)** SROC curve for POC-CCA with trace results considered negative (T−); **(c)** SROC curve for PCR; SROC curve for PCR-ELISA.

Multivariate analyses could only be conducted for POC-CCA (with trace results interpreted as positive or negative), conventional PCR, and PCR-ELISA (Figure 4). Conventional PCR showed the highest sensitivity (91.5%; 95% CI: 78.9-96.8), whereas POC-CCA (T-) and PCR showed the highest specificity (88%; 95% CI: 77-95; 86.9 95% CI: 57.7-97.0, respectively). Accuracy ranged from 70% (95% CI: 61-78) for POC-CCA (T+) to 82% (95% CI: 70-92) for PCR. The highest DOR was observed for PCR (99.4; 95% CI: 24.2-407.8), followed by PCR-ELISA (12.4; 95% CI: 0.67-229.08). Both POC-CCA reading criteria yielded lower DOR values ( 7.14 (95% CI 1.86-20.41) for T+ and 5.96 95% CI: 1.95-18.18 for T-). Overall, PCR showed the highest diagnostic performance, with a positive likelihood ratio (LR+) of 6.96 and an LR− of 0.09, indicating good ability to both confirm and exclude disease. In contrast, POC-CCA showed only moderate rule-in capacity (LR+ ~3-4) and limited rule-out value (LR− ~0.46), suggesting limited clinical impact. PCR-ELISA did not demonstrate a clear advantage, with imprecise LRs and CIs crossing unity (Figure 4).

### Risk of bias assessment

Risk of bias, assessed with QUADAS-2, varied across domains (Supplementary File 7 [S7 File]). Most studies were rated as low risk of bias for flow and timing and for patient selection. However, patient selection also had a substantial proportion of unclear risk, primarily due to insufficient reporting of participant inclusion criteria. In the index test domain, unclear risk predominated, largely due to a lack of information on blinding. All studies were rated as unclear risk in the reference standard domain, reflecting the limitations of parasitological methods as the gold standard. Most studies were rated as low risk for applicability, indicating general alignment with the review question.

## DISCUSSION

This systematic review and meta-analysis synthesized the available evidence on the performance of various diagnostic methods for *S. mansoni* infection in Brazil, highlighting notable differences between serological and molecular approaches. The findings confirm substantial heterogeneity among studies, which limits the direct generalization of results across different epidemiological settings. Evaluating diagnostic test performance is crucial for understanding their clinical utility, that is, their ability to reliably inform treatment and management decisions. Traditionally, this evaluation is based on metrics such as sensitivity and specificity, which reflect, respectively, the proportion of true-positive cases correctly identified, and the proportion of healthy individuals accurately classified. However, the isolated interpretation of these measures has limitations: sensitivity and specificity are intrinsically interdependent, being linked through the diagnostic threshold and the underlying disease spectrum. Any change in the positivity criterion of a test typically results in a trade-off between these parameters. Therefore, interpreting these measures in isolation may be misleading, particularly when comparing studies that differ in threshold definition, population characteristics, or infection intensity.

For this reason, diagnostic performance in this review was conceptualized based on the joint behavior of sensitivity and specificity rather than on single summary indicators. Whenever sufficient data were available (≥ 4 studies per comparison), we applied a bivariate random-effects model, which simultaneously estimates pooled sensitivity and specificity while explicitly modeling the correlation between them. This approach recognizes that the two measures arise from the same 2 × 2 contingency framework and are statistically dependent.

The bivariate framework also incorporates between-study heterogeneity at two levels: variability in true sensitivity and specificity across studies, and variability in their covariance structure. By allowing both parameters to vary randomly while preserving their correlation, the model provides a more coherent and methodologically appropriate synthesis than separate univariate pooling approaches.

Defining the reference standard was a central methodological decision in this study. Most investigations used parasitological methods-predominantly Kato-Katz-as the reference standard for the diagnosis of *S. mansoni* infection due to its high specificity, operational feasibility, and field applicability. However, it is widely recognized as an imperfect reference standard in low-intensity settings because of its limited sensitivity.

 This limitation has direct implications for diagnostic accuracy studies. When the reference standard fails to detect true infections, more analytically sensitive tests (e.g., molecular or antigen-detection assays) may appear to generate false positives, leading to an artificial underestimation of specificity. This phenomenon, known as imperfect gold standard bias, may distort comparative interpretations between tests and contribute to heterogeneity across studies.

The impact of this limitation is further amplified by variability in the implementation of parasitological methods (e.g., number of stool samples and number of slides examined), information that was frequently incompletely or inconsistently reported. Although stratified analyses or composite reference standards were considered as mitigation strategies, these could not be robustly implemented due to insufficient reporting. For this reason, the QUADAS-2 reference standard domain was conservatively rated as unclear, and the potential implications of differential misclassification bias are explicitly acknowledged in the interpretation of the findings.

Importantly, within individual studies, the same reference standard was applied to all index tests. Therefore, although absolute accuracy estimates may be affected, intra-study comparisons remain internally consistent, and bias is more likely to influence cross-study comparisons than within-study contrasts.

 ELISA-based serological tests showed moderate performance, with high sensitivity, particularly for SWAP IgG (> 90%), but limited specificity (66%). This profile suggests greater value for population screening than for confirmatory diagnosis. International reviews report similar findings and highlight antigen heterogeneity and the lack of assay standardization as key barriers to comparability and robust evidence^19^ ^,^ ^31^.

POC-CCA performance depended strongly on the cut-off. Interpreting trace results as positive (T+) increased sensitivity versus T- (66% vs 44%) but reduced specificity (44% vs 88%). Similar findings in Brazilian studies^9^ ^,^ ^20^ ^,^ ^61^ underscore the difficulty of standardizing field interpretation criteria, particularly in low-endemicity settings where false positives can undermine epidemiological surveillance.

Molecular tests, including PCR and real-time PCR, showed the best overall performance, with sensitivity and specificity exceeding 80%. These findings support evidence from international reviews^24^ ^,^ ^27^, which highlight the potential of molecular methodologies as an alternative reference standard in low-parasite-load settings. However, the Brazilian Unified Health System (*Sistema Único de Saúde* - SUS) is constrained by heterogeneity, limited evidence, and high operational costs. LAMP showed lower sensitivity than all other molecular methods evaluated; however, only two studies assessing this technique were included, one of which had only 13 patients with infection^25^.

The heterogeneity observed across most pooled analyses appears to be driven by identifiable methodological and epidemiological factors that differ according to each diagnostic category. For ELISA-based assays (SEA and SWAP), heterogeneity was particularly pronounced (I² > 80%), likely reflecting multiple structured differences across studies. Even within the same antigen category, preparation methods varied, including in-house crude extracts, batch variability, and purification procedures. Antibody isotypes also differed, with studies measuring IgG, IgM, or subclasses, which directly influences the sensitivity-specificity balance. Moreover, cutoffs were not standardized and often relied on study-specific receiver operating characteristic (ROC) analyses or arbitrary optical density thresholds. Together with differences in the reference standard and infection intensity across populations, these factors likely explain the wide dispersion of performance estimates. Overall, heterogeneity in ELISA meta-analyses appears to be predominantly driven by assay standardization issues and threshold effects, compounded by variability in population spectra and comparator methods.

For POC-CCA, heterogeneity remained even after stratifying analyses by trace interpretation. Classifying a “trace” band is itself a threshold decision, and differences in visual reading, operator training, and field conditions may have contributed to inconsistent classification. Variation in endemicity and parasite burden across study sites also affects circulating antigen levels and, in turn, test sensitivity and specificity under different positivity criteria. Differences in the intensity of parasitological reference testing, such as the number of Kato-Katz slides examined, likely added further variability. Overall, heterogeneity in POC-CCA analyses appears to be driven primarily by threshold effects, epidemiological context, and the limited sensitivity of the comparator.

Conventional PCR demonstrated high pooled sensitivity but remained heterogeneous across studies. This variability likely reflects differences in sample type, DNA extraction methods, target sequences, amplification protocols, and positivity criteria such as cycle thresholds. Given that molecular detection is highly sensitive to technical parameters, even small methodological differences can substantially affect performance. Additionally, the low sensitivity of the parasitological reference standard in low-intensity settings may have produced apparent false positives, particularly in low-endemicity areas. Thus, heterogeneity in PCR meta-analyses appears to be driven largely by technical diversity and misclassification from the reference standard.

PCR-ELISA analyses exhibited wide CIs and unstable DOR estimates. This likely reflects the limited number of available studies and methodological differences across hybridization detection platforms and extraction protocols. Differences in endemicity and limited sample sizes further reduced the stability of estimates. In this subgroup, heterogeneity appears to result from both technical diversity and limited statistical power.

Only two studies assessed LAMP, and one included a small number of infected participants. The observed variability is therefore likely influenced by sample size instability and differences in primer sets, amplification targets, and implementation conditions between laboratory and field settings. In early-stage evaluations, such methodological heterogeneity is expected and may not yet reflect stable performance.

In contrast, the TF-Test meta-analysis yielded I² = 0%; however, this estimate was based on only two studies from similar epidemiological settings, limiting the statistical power to detect heterogeneity. Therefore, the absence of statistical heterogeneity should not be interpreted as proof of methodological uniformity.

Across comparisons, heterogeneity likely reflected non-standardized use of the parasitological reference standard, lack of composite reference standards in low-intensity settings, inconsistent reporting of infection intensity and endemicity, and differences in study populations. Additionally, differences in pooling strategies may have influenced the summary estimates.

Overall, the high heterogeneity likely reflects genuine clinical and methodological diversity rather than random statistical fluctuation. These findings underscore the need for more standardized diagnostic protocols, clearer threshold criteria, and improved reference frameworks to improve comparability and strengthen the evidence base for schistosomiasis control and elimination strategies.

Kato-Katz was the main reference standard, but its implementation varied widely across studies in the number of stool samples examined, the number of slides examined, and occasional combination with other parasitological methods. Given that the sensitivity of Kato-Katz is directly influenced by parasite burden and sampling intensity^62^, this variability likely introduced differential misclassification, particularly in low-intensity infections, and increased between-study variation in both sensitivity and specificity.

Epidemiological context also varied markedly across Brazilian regions, from higher-burden areas to low-endemicity or post-control settings. Studies were conducted in areas with heterogeneous transmission patterns and varying infection intensities, ranging from higher-burden settings to low-endemicity or post-control contexts. Diagnostic performance, especially for parasitological and antigen-detection methods, is highly dependent on parasite load. In high-transmission areas, egg output tends to be higher and more consistently detected, whereas in low-intensity settings, intermittent egg excretion increases the likelihood of false-negative parasitological results. Because prevalence and infection intensity were inconsistently reported and could not be systematically incorporated into stratified analyses, spectrum effects likely contributed substantially to heterogeneity.

Threshold effects were another major source of variability. The distinction between trace-positive and trace-negative POC-CCA interpretations exemplifies how threshold variation directly affects sensitivity-specificity trade-offs. Likewise, although conventional PCR demonstrated the highest pooled sensitivity and DOR in the bivariate analysis, the wide CIs-particularly for PCR-ELISA-indicate imprecision. ELISA-based assays also varied in antigen source, antibody isotype, and cut-off definitions, while molecular methods differed in target sequences, amplification protocols, extraction procedures, and positivity criteria. As positivity thresholds and protocols were not consistently standardized or reported, explicit modeling was not feasible and likely contributed to dispersion in pooled estimates and SROC plots.

The study population also differed, including community-based surveys and clinically suspected or symptomatic groups. Variation in age, exposure, and case mix likely contributed to spectrum effects, particularly in serological assays, where immune response intensity may vary with infection chronicity and reinfection history.

Additionally, methodological diversity within test categories limited comparability across studies. For example, “PCR” encompassed different biological samples (stool versus urine), DNA extraction protocols, and platforms, meaning that studies grouped under the same label did not necessarily evaluate identical technological implementations. Similar variability was observed for immunological assays and antigen-detection methods.

Finally, several pooled comparisons were based on a small number of studies, reducing statistical stability and limiting the ability to formally explore heterogeneity through meta-regression analyses. In some instances, the apparent absence of heterogeneity (e.g., I² = 0%) likely reflects limited statistical power rather than true methodological homogeneity.

Taken together, the observed heterogeneity was multifactorial and reflects broader structural challenges in diagnostic accuracy research for schistosomiasis in endemic settings. QUADAS-2 identified important limitations in patient selection, index test performance, and the reference standard, which was typically limited to Kato-Katz. Given the known limitations and inconsistent implementation of Kato-Katz, the true risk of bias may approach high risk due to potential differential misclassification. This is particularly relevant when interpreting potential underestimation of specificity in more sensitive index tests, as well as heterogeneity and inconsistency across studies. These limitations reflect the complexity of evaluating emerging diagnostic methods in endemic settings and highlight the need for greater methodological rigor and transparency in future diagnostic accuracy studies.

Overall, the evaluated tests did not meet the minimum WHO target product profile requirements for schistosomiasis control and elimination programs. The tests described failed to achieve the minimum and ideal characteristics in terms of sensitivity, specificity, time to result, field applicability, and cost. The sensitivities obtained were below the reference values for most tests (minimum > 80% for 50 individuals or > 60% for 100 individuals; ideal > 78% and > 75%, respectively), which implies a higher risk of false negatives and, consequently, failure to correctly identify infected individuals. Similarly, specificity fell below the acceptable thresholds for most tests (minimum > 97% for 50 individuals or > 95% for 100 individuals; ideal > 98.5% and > 96.5%, respectively), compromising test reliability and increasing the likelihood of false positives. In addition, the time required to obtain results exceeded the maximum acceptable limit of 2 h and failed to meet the preferred target of less than 30 min. Together, these limitations restrict the clinical utility of the evaluated methods relative to current international quality standards^63^.

This review has certain limitations. First, the evidence was concentrated in Minas Gerais, whereas other historically hyperendemic states were not represented. As these regions still bear substantial disease burden and ongoing transmission, particularly in rural and riverside communities, the available evidence may not capture the full national epidemiological landscape^5^. This distribution gap reinforces the need for multicenter studies across a broader range of endemic settings. 

Second, stratified analyses by schistosomiasis prevalence was not possible as most studies did not report prevalence. This is relevant because diagnostic accuracy varies by epidemiological context: higher parasite burdens can improve the sensitivity of parasitological methods, whereas low-endemicity settings-now increasingly common in Brazil-have a greater risk of false negatives^16^ ^,^ ^18^. This limitation may reduce the applicability of the findings to specific surveillance and disease elimination strategies.

Third, reliance on Kato-Katz as the reference standard across studies is a limitation, as its sensitivity is strongly influenced by the number of stool samples and slides examined. Thus, studies based on a single sample or a few slides are more likely to underestimate true parasite burden, particularly in low-endemicity settings. This weakens comparability between studies and may bias accuracy estimates for the evaluated tests^63^. Despite these limitations, this review provides a useful basis for comparative assessment of diagnostic methods for *S. mansoni* infection in Brazil. 

From a practical standpoint, our findings highlight the need for a combined diagnostic approach, tailored to different epidemiological settings, the commercial availability of tests, and the existing infrastructure. Although limited in sensitivity, the Kato-Katz method remains essential as a basic epidemiological tool. Serological tests can play an important role in rapid screening, while molecular techniques emerge as a future reference for diagnostic confirmation and monitoring in areas progressing toward transmission elimination. WHO recommendations indicate that in low-endemicity or elimination-phase areas, the use of more sensitive methods and the adoption of two-step diagnostic strategies, with a screening test followed by a confirmatory test, are crucial to prevent both under-detection of cases and excessive false positives^3^ ^,^ ^64^ ^-^ ^65^. 

In summary, this meta-analysis confirms that molecular tests, particularly conventional PCR, exhibit the highest diagnostic performance, while serological methods provide useful alternatives but remain limited by lack of standardization and result variability. The implementation of combined diagnostic strategies, together with multicenter and methodologically robust studies that incorporate economic evaluations, is essential to support surveillance, control, and elimination policies for *S. mansoni* infection in Brazil.

## Data Availability

Data available upon request.

## References

[1] Fundação Oswaldo Cruz (2022). Recomendações dos membros do Programa de Pesquisa Translacional em Esquistossomose da Fundação Oswaldo Cruz (Fio Schisto) para o controle e eliminação da esquistossomose humana no Brasil.

[2] Brasil. Ministério da Saúde. Secretaria de Vigilância em Saúde e Ambiente. Departamento de Doenças de Condições Crônicas e Infecções Sexualmente Transmissíveis (2024). Vigilância da esquistossomose mansoni: diretrizes técnicas.

[3] World Health Organization (WHO) (2022). WHO guideline on control and elimination of human schistosomiasis.

[4] Li Q, Li Y, Guo Y, Li S, Wang Q, Lin W (2025). Global trends of schistosomiasis burden from 1990 to 2021 across 204 countries and territories: Findings from GBD 2021 Study. Acta Tropica.

[5] Santos MIF, Teles RS, Rocha CJ, Silva TO, Gonçalves MML (2023). Mortality due to neglected tropical diseases in Brazil: spatial and temporal analysis. PLoS Neglected Tropical Diseases.

[6] Paiva MC, Chemello FB, Rocha IG, Domingos ET, França RF, Reda K (2025). Epidemiologia das internações por esquistossomose no Brasil nos últimos 10 anos (2014-2024). Braz J Implantol Health Sci.

[7] Santos CTJ, Oliveira LJS, Ribeiro AL, Reis MC, Castro RS, Almeida MCC (2025). Mortality due to schistosomiasis in an endemic area of Brazil: a population-based ecological study. BMC Public Health.

[8] Ponzo E, Duarte M, Amaral M, Costa C, Pinto P (2024). Insights into the epidemiology, pathogenesis, and control of schistosomiasis. Microorganisms.

[9] Grenfell RFQ, Pedrosa ML, Couto FFB, Oliveira ÁA, Coelho PMZ, Katz N (2019). Suitability of commercially available POC-CCA tests for schistosomiasis: Considerations for efficiency, reproducibility and decision-making criteria for field application in areas of low endemicity. J Immunol Methods.

[10] Vaillant MT (2024). Diagnostic tests for human Schistosoma mansoni and the importance of accurate diagnosis for surveillance, control, and elimination. The Lancet Microbe.

[11] Fasogbon IV, Nyakundi EO, Tusubira D, Ogbonnia EC, Ashley J, Sasikumar S (2024). A critical review of the limitations of current diagnostic techniques for schistosomiasis. All Life.

[12] World Health Organization (2021). Ending the neglect to attain the Sustainable Development Goals: roadmap for neglected tropical diseases 2021-2030.

[13] Katz N, Chaves A, Pellegrino J (1972). A simple device for quantitative stool thick-smear technique in Schistosoma mansoni. Rev Inst Med Trop São Paulo.

[14] Caldeira K, Teixeira CF, Silveira MB, Fries LCC, Romanzini J, Bittencourt HR (2012). Comparison of the Kato-Katz and Helmintex methods for the diagnosis of schistosomiasis in a low-intensity transmission focus in Bandeirantes, Paraná, southern Brazil. Mem Inst Osw Cruz.

[15] Carneiro KJSG, Carneiro CS, Carneiro CS (2012). Improving diagnosis of schistosomiasis. Rev Soc Bras Med Trop.

[16] Lindholz CG, Favero V, De Marco Veríssimo C, Russo Frasca Candido R, De Souza RP, Dos Santos RR (2018). Study of diagnostic accuracy of Helmintex, Kato-Katz, and POC CCA methods for diagnosing intestinal schistosomiasis in Candeal, a low intensity transmission area in northeastern Brazil. PLoS Negl Trop Dis.

[17] Oliveira WJ, Magalhães FC, Elias AM, Castro VN, Favero V, Lindholz CG (2018). Evaluation of diagnostic methods for the detection of intestinal schistosomiasis in endemic areas with low parasite loads: Saline gradient, Helmintex, Kato-Katz and rapid urine test. PLoS Negl Trop Dis.

[18] Oliveira EJ, Kanamura HY, Lima DMC (2005). Efficacy of an enzyme-linked immunosorbent assay as a diagnostic tool for schistosomiasis mansoni in individuals with low worm burden. Mem Inst Osw Cruz.

[19] Ramos LMS, Pereira DSC, Oliveira LV, Graeff-Teixeira C (2024). Accuracy of commercial ELISA and ICT for screening schistosomiasis infections at a low endemicity area in Brazil. Transactions of The Trans R Soc Trop Med Hyg.

[20] Bezerra DF, Pinheiro MCC, Barbosa L, Viana AG, Fujiwara RT, Bezerra FSM (2021). Diagnostic comparison of stool exam and point-of-care circulating cathodic antigen (POC-CCA) test for schistosomiasis mansoni diagnosis in a high endemicity area in northeastern Brazil. Parasitology.

[21] Carvalho GC, Marques LHS, Gomes LI, Rabello A, Ribeiro LC, Scopel KKG (2012). Polymerase chain reaction for the evaluation of Schistosoma mansoni infection in two low endemicity areas of Minas Gerais, Brazil. Mem Inst Osw Cruz.

[22] Magalhães FC, Resende SD, Senra C, Graeff-Teixeira C, Enk MJ, Coelho PMZ (2020). Accuracy of real-time polymerase chain reaction to detect Schistosoma mansoni-infected individuals from an endemic area with low parasite loads. Parasitology.

[23] Siqueira LVM, Gomes LI, Oliveira E, Oliveira ER, Oliveira AA, Enk MJ, Carneiro NF, Rabello A, Coelho PMZ (2015). Evaluation of parasitological and molecular techniques for the diagnosis and assessment of cure of schistosomiasis mansoni in a low transmission área. Mem Inst Oswaldo Cruz.

[24] Li HM, Qin ZQ, Bergquist R, Qian MB, Xia S, Lv S, Xiao N (2021). Nucleic acid amplification techniques for the detection of Schistosoma mansoni infection: a structured review and meta-analysis. Int J Infec Dis.

[25] Gandasegui J, Fernández Soto P, Muro A, Simões Barbosa C, Lopes de Melo F, Loyo R (2018). A field survey using LAMP assay for detection of Schistosoma mansoni in a low transmission area of schistosomiasis in Umbuzeiro, Brazil: Assessment in human and snail samples. PLoS Negl Trop Dis.

[26] Gomes ECS, Barbosa WL, De Melo FL (2022). Evaluation of SmITS1-LAMP performance to diagnosis schistosomiasis in human stool samples from an endemic area in Brazil. Exper Parasitol.

[27] Zhou X, Li J, Qiu J, Feng T, Lv C, Deng W (2025). Test accuracy of loop-mediated isothermal amplification for schistosomiasis in low endemicity areas: a systematic review and meta-analysis. Infec Dis Pov.

[28] Frota SM, Carneiro TR, Queiroz JAN, Alencar LM, Heukelbach J, Bezerra FSM (2011). Combination of Kato-Katz faecal examinations and ELISA to improve accuracy of diagnosis of intestinal schistosomiasis in a low-endemic setting in Brazil. Acta Tropica.

[29] Oliveira LMA, Santos HLC, Gonçalves MML, Barreto MGM, Peralta JM (2010). Evaluation of polymerase chain reaction as an additional tool for the diagnosis of low-intensity Schistosoma mansoni infection. Diagn Microbiol Infect Dis.

[30] Oyeyemi OT, Corsini CA, Gonçalves G, Borges WDC, Grenfell RFQ (2021). Evaluation of schistosomula crude antigen (SCA) as a diagnostic tool for Schistosoma mansoni in low endemic human population. Scientific Reports.

[31] Feleke DG, Alemu Y, Bisetegn H, Debash H (2023). Accuracy of diagnostic tests for detecting Schistosoma mansoni and S. haematobium in sub-Saharan Africa: a systematic review and meta-analysis. BioMed Res Int.

[32] Buonfrate D, Ferrari TCA, Akim Adegnika A, Stothard JR, Gobbi FG (2025). Human schistosomiasis. The Lancet.

[33] Cochrane JP, Bossuyt PM, Davenport C, Deeks JJ, Hyde C, Leeflang MM (2019). Cochrane Handbook for Systematic Reviews of Diagnostic Test Accuracy Version 1.0.0.

[34] Page MJ, Moher D, Bossuyt PM, Boutron I, Hoffmann TC, Mulrow CD (2021). PRISMA 2020 explanation and elaboration: updated guidance and exemplars for reporting systematic reviews. Br Med J.

[35] Haynes RB, Wilczynski NL (2004). Optimal search strategies for retrieving scientifically strong studies of diagnosis from Medline: analytical survey. Br Med J.

[36] Mendeley (2023). Reference Manager-Mendeley.

[37] Ouzzani M, Hammady H, Fedorowicz Z, Elmagarmid A (2016). Rayyan-a web and mobile app for systematic reviews. Systematic Reviews.

[38] Chu H, Cole SR (2006). Bivariate meta-analysis of sensitivity and specificity with sparse data: a generalized linear mixed model approach. J Clin Epidemiol.

[39] Plana MN, Arevalo-Rodriguez I, Fernández-García S, Soto J, Fabregate M, Pérez T (2022). Meta-DiSc 2.0: a web application for meta-analysis of diagnostic test accuracy data. BMC Med Res Methodo.

[40] Chu H, Cole SR (2006). Bivariate meta-analysis of sensitivity and specificity with sparse data: a generalized linear mixed model approach. J Clin Epidemiol.

[41] Fekete JT, Győrffy B (2025). MetaAnalysisOnline.com: Web-Based Tool for the Rapid Meta-Analysis of Clinical and Epidemiological Studies. J Med Internet Res.

[42] Whiting PF, Rutjes AWS, Westwood ME, Mallett S, Deeks JJ, Reitsma JB (2011). QUADAS-2: a revised tool for the quality assessment of diagnostic accuracy studies. Ann Intern Med.

[43] Enk MJ, Silva GO, Rodrigues NB (2012). Diagnostic accuracy and applicability of a PCR system for the detection of Schistosoma mansoni DNA in human urine samples from an endemic area. PLoS ONE.

[44] Pontes LA, Oliveira MC, Katz N, Dias-Neto E, Rabello A (2003). Comparison of a polymerase chain reaction and the Kato-Katz technique for diagnosis of infection with Schistosoma mansoni. Mem Inst Oswaldo Cruz.

[45] Gonçalves MML, Barreto MGM, Peralta RHS, Gargioni C, Gonçalves T, Igreja RP (2006). Immunoassays as an auxiliary tool for the serodiagnosis of Schistosoma mansoni infection in individuals with low intensity of egg elimination. Acta Tropica.

[46] Gomes LI, Marques LHS, Enk MJ, Coelho PMZ, Rabello A (2009). Further evaluation of an updated PCR assay for the detection of Schistosoma mansoni DNA in human stool samples. Mem Inst Oswaldo Cruz.

[47] Gomes LI, Marques LHS, Enk MJ, Oliveira MC, Coelho PMZ, Rabello A (2010). Development and evaluation of a sensitive PCR-ELISA system for detection of Schistosoma infection in feces. Mem Inst Oswaldo Cruz.

[48] Siqueira LMV, Coelho PMZ, Oliveira ÁA, Massara CL, Carneiro NFF, Lima ACL (2011). Evaluation of two coproscopic techniques for the diagnosis of schistosomiasis in a low-transmission area in the state of Minas Gerais, Brazil. Mem Inst Oswaldo Cruz.

[49] Coelho PMZ, Siqueira LMV, Grenfell RFQ, Almeida NBF, Katz N, Almeida Á (2016). Improvement of POC CCA interpretation by using lyophilization of urine from patients with Schistosoma mansoni low worm burden: Towards an elimination of doubts about the concept of trace. PLoS Negl Trop Dis.

[50] Siqueira LM, Couto FF, Taboada D, Oliveira ÁA, Carneiro NF, Oliveira E (2016). Performance of POC-CCA® in diagnosis of schistosomiasis mansoni in individuals with low parasite burden. Rev Soc Bras Med Trop.

[51] Ferreira FT, Fidelis TA, Pereira TA, Otoni A, Queiroz LC, Amâncio FF, Antunes CM (2017). Sensitivity and specificity of the circulating cathodic antigen rapid urine test in the diagnosis of Schistosomiasis mansoni infection and evaluation of morbidity in a low endemic area in Brazil. Rev Soc Bras Med Trop.

[52] Nacife MBPSL, Siqueira LMV, Martins R, Vianna VN, Barbosa KF, Masioli CZ (2018). Prevalence of schistosomiasis mansoni in indigenous Maxakali villages, Minas Gerais, Brazil. Rev Inst Med Trop São Paulo.

[53] Queiroz RFQ, Harn D, Coelho PMZ (2018). Overcoming challenges in the diagnosis of Schistosoma mansoni infections using POC tests, recombinant protein and monoclonal antibody technologies. FASEB Journal.

[54] Senra C, Gomes LI, Siqueira LMV, Coelho PMZ, Rabello A, Oliveira E (2018). Development of a laboratorial platform for diagnosis of schistosomiasis mansoni by PCR-ELISA. BMC Research Notes.

[55] Souza SRM, Nogueira JFC, Dias IHL, Fonseca ALS, Favero V, Geiger SM (2020). The use of the circulating cathodic antigen (CCA) urine cassette assay for the diagnosis and assessment of cure of Schistosoma mansoni infections in an endemic area of the Amazon region. Rev Soc Bras Med Trop.

[56] Siqueira LMV, Senra C, De Oliveira ÁA, Carneiro NF, Gomes LI, Rabello A, Coelho PMZ (2021). A real-time PCR assay for the diagnosis of intestinal schistosomiasis and cure assessment after the treatment of individuals with low parasite burden. Front Immunol.

[57] Sousa SRM, Dias IHL, Fonseca ALS, Contente BR, Nogueira JFC, Oliveira TNC (2019). Concordance of the point-of-care circulating cathodic antigen test for the diagnosis of intestinal schistosomiasis in a low endemicity area. Infect Dis Poverty.

[58] Oyeyemi OT, Corsini CA, Gonçalves G, de Castro Borges W, Grenfell RFQ (2021). Evaluation of schistosomula crude antigen (SCA) as a diagnostic tool for Schistosoma mansoni in low endemic human population. Sci Rep.

[59] Magalhães FDC, Moreira JMP, Rezende MCF, Favero V, Graeff-Teixeira C, Coelho PMZ (2023). Evaluation of isotype-based serology for diagnosis of Schistosoma mansoni infection in individuals living in endemic areas with low parasite burden. Acta Tropica.

[60] Lopes KF, Freire ML, Lima DCS, Enk MJ, Oliveira EJ, Geiger SM (2023). Development and evaluation of an indirect ELISA using a multiepitope antigen for the diagnosis of intestinal schistosomiasis. Parasitology.

[61] Pieri OS, Bezerra FSM, Coelho PMZ, Enk MJ, Favre TC, Graeff-Teixeira C (2023). Accuracy of the urine point-of-care circulating cathodic antigen assay for diagnosing Schistosomiasis mansoni infection in Brazil: A multicenter study. Rev Soc Bras Med Trop.

[62] Carneiro TR, Pinheiro MCC, Oliveira SM, Hanemann ALP, Queiroz JAN, Bezerra FSM (2012). Aumento da detecção da esquistossomose com Kato-Katz e SWAP-IgG-ELISA, em área de baixa endemicidade, no nordeste do Brasil. Rev Soc Bras Med Trop.

[63] Berhe N, Medhin G, Erko B, Smith T, Gedamu S, Bereded D (2004). Variations in helminth faecal egg counts in Kato-Katz thick smears and their implications in assessing infection status with Schistosoma mansoni. Acta Tropica.

[64] World Health Organization (WHO) (2021). Diagnostic target product profiles for monitoring, evaluation and surveillance of schistosomiasis control programmes.

[65] World Health Organization (2022). WHO guideline on control and elimination of human schistosomiasis.

