# Erratum: Accuracy of diagnostic strategies for detecting Schistosoma mansoni infection in Brazil: A systematic review and meta-analysis

**DOI:** 10.1590/0037-8682-0466b-2025

**Published:** 2026-09-14

**Revista da Sociedade Brasileira de Medicina Tropical/Journal of the Brazilian Society of Tropical Medicine**

 In the document: Assis TSM, Freire ML, Silva SN, Avelar DM, Cota G, Rabello A. Accuracy of diagnostic strategies for detecting Schistosoma mansoni infection in Brazil: A systematic review and meta-analysis. Rev Soc Bras Med Trop. 2026 Aug 3;59:e04662025. doi: 10.1590/0037-8682-0466-2025. PMID: 42561331; PMCID: PMC13432800 

**Page 11/13 (ACKNOWLEDGMENTS)**

 We are grateful for all the support and endorsement of the Brazilian Ministry of Health, specifically the General Coordination of Management of Clinical Protocols and Therapeutic Guidelines CGPCDT) and Instituto René Rachou (Fundação Oswaldo Cruz).

**Is correct**

We are grateful for all the support and endorsement of Instituto René Rachou (Fundação Oswaldo Cruz) and Programa Inova Fiocruz.

