# Supplementary material for: Accuracy of diagnostic strategies for detecting Schistosoma mansoni infection in Brazil: A systematic review and meta-analysis
Source: Rev Soc Bras Med Trop. 2026 Aug 3;59:e0466-2025. doi: 10.1590/0037-8682-0466-2025 (PMC13432800; doi:10.1590/0037-8682-0466-2025)
Supplement: Supplementary File 1 (S1 File) [file 1678-9849-rsbmt-59-e0466-2025-md1.pdf]

S1 Table. Search strategy used in each database

**Database**	**Search strategy**	**N° of retrieved documents**
MEDLINE (by Pubmed)	**Population:**<br>**#1** (((((((((((((((Schistosomiasis mansoni[MeSH Terms]) OR (Schistosomiasis mansoni[Title/Abstract])) OR (Schistosoma mansoni Infection[Title/Abstract])) OR (Infection, Schistosoma mansoni[Title/Abstract])) OR (Infections, Schistosoma mansoni[Title/Abstract])) OR (Schistosoma mansoni Infections[Title/Abstract])) OR (Schistosomiasis, Intestinal[Title/Abstract])) OR (Intestinal Schistosomiases[Title/Abstract])) OR (Intestinal Schistosomiasis[Title/Abstract])) OR (Schistosomiases, Intestinal[Title/Abstract])) OR (Schistosomiasis[MeSH Terms])) OR (Schistosomiasis[Title/Abstract])) OR (Schistosomiases[Title/Abstract])) OR (Schistosoma Infection[Title/Abstract])) OR (Infection, Schistoma[Title/Abstract])))	494<br>(10/01/2024)
	**Intervention:**<br>#2<br>(((Diagnosis[MeSH Terms]) OR (Diagnosis[Title/Abstract])) OR (Diagnoses[Title/Abstract])) OR (Diagnose[Title/Abstract]))) OR (Serologic Tests[MeSH Terms])) OR (Serologic Tests[Title/Abstract])) OR (Serologic Test[Title/Abstract])) OR (Test, Serologic[Title/Abstract])) OR (Tests, Serologic[Title/Abstract])) OR (Serological Tests[Title/Abstract])) OR (Serological Test[Title/Abstract])) OR (Test, Serological[Title/Abstract])) OR (Tests, Serological[Title/Abstract])) OR (Serodiagnosis[Title/Abstract])) OR (Serodiagnoses[Title/Abstract])) OR (Fluorescent Antibody Technique[MeSH Terms])) OR (Fluorescent Antibody Technique[Title/Abstract])) OR (Antibody Technique, Fluorescent[Title/Abstract])) OR (Antibody Techniques, Fluorescent[Title/Abstract])) OR (Immunofluorescence Technique[Title/Abstract])) OR (Immunofluorescence Techniques[Title/Abstract])) OR (Immunofluorescence[Title/Abstract])) OR (Enzyme-Linked Immunosorbent Assay[MeSH Terms])) OR (Enzyme-Linked Immunosorbent Assay[Title/Abstract])) OR (Assay, Enzyme-Linked Immunosorbent[Title/Abstract])) OR (Assays, Enzyme-Linked Immunosorbent[Title/Abstract])) OR (Enzyme Linked Immunosorbent Assay[Title/Abstract])) OR (Enzyme-Linked Immunosorbent Assays[Title/Abstract])) OR (Immunosorbent	

	Assay, Enzyme-Linked[Title/Abstract])) OR (Immunosorbent Assays, Enzyme-Linked[Title/Abstract])) OR (ELISA[Title/Abstract])) OR (Rapid Diagnostic Tests[MeSH Terms])) OR (Rapid Diagnostic Tests[Title/Abstract])) OR (Diagnostic Test, Rapid[Title/Abstract])) OR (Diagnostic Tests, Rapid[Title/Abstract])) OR (Rapid Diagnostics[Title/Abstract])) OR (Diagnostic, Rapid[Title/Abstract])) OR (Diagnostics, Rapid[Title/Abstract])) OR (Rapid Diagnostic[Title/Abstract])) OR (Rapid Diagnostic Test[Title/Abstract])) OR (Molecular Diagnostic Techniques[MeSH Terms])) OR (Molecular Diagnostic Techniques[Title/Abstract])) OR (Diagnostic Technique, Molecular[Title/Abstract])) OR (Diagnostic Techniques, Molecular[Title/Abstract])) OR (Molecular Diagnostic Technique[Title/Abstract])) OR (Molecular Testing[Title/Abstract])) OR (Testing, Molecular[Title/Abstract])) OR (Testing, Molecular Diagnostic[Title/Abstract])) OR (Polymerase Chain Reaction[MeSH Terms])) OR (Polymerase Chain Reaction[Title/Abstract])) OR (Polymerase Chain Reactions[Title/Abstract])) OR (Reaction, Polymerase Chain[Title/Abstract])) OR (PCR[Title/Abstract])) OR (LAMP assay[Title/Abstract])) OR (loop-mediated isothermal amplification technique[Title/Abstract])) OR (LAMP technique[Title/Abstract]) OR (LAMP[Title/Abstract])	
	**Outcome:**<br>**#3** ((sensitiv*[Title/Abstract] OR sensitivity and specificity[MeSH Terms] OR diagnose[Title/Abstract] OR diagnosed[Title/Abstract] OR diagnoses[Title/Abstract] OR diagnosing[Title/Abstract] OR diagnosis[Title/Abstract] OR diagnostic[Title/Abstract] OR diagnosis[MeSH:noexp] OR (diagnostic equipment[MeSH:noexp] OR diagnostic errors[MeSH:noexp] OR diagnostic imaging[MeSH:noexp] OR diagnostic services[MeSH:noexp]) OR diagnosis, differential[MeSH:noexp] OR diagnosis[Subheading:noexp])) OR ((specificity[Title/Abstract]))	
	**Location:**<br>**#4** (((((Brazil[MeSH Terms])) OR (Brazil)) OR (Brazilian)))<br><br>**#4** #1 AND #2 AND #3	
EMBASE	**Population:**<br>**#1** ('schistosomiasis mansoni'/exp OR 'manson schistosomiasis' OR 's. mansoni infection' OR 's. mansoni infections' OR 'schistosoma mansoni infection' OR 'schistosoma mansoni infections' OR 'infection by schistosoma mansoni' OR 'infection of schistosoma mansoni' OR 'mansoni schistosomiasis' OR	1.045<br>(10/01/2024)

	'schistosomiasis due to schistosoma mansoni' OR 'schistosomiasis mansoni' OR 'schistosomiasis'/exp OR 'schistosoma infection' OR 'schistosoma infections' OR 'bilharzioses' OR 'infection by schistosoma' OR 'schistomiasis' OR 'schistosomatosis' OR 'schistosome infection' OR 'schistosomiases' OR 'schistosomiasis' OR 'schistosomosis' OR 'snail fever')
	**Intervention:**<br>**#2** ('diagnosis'/exp OR 'bacteriologic diagnosis' OR 'diagnosis' OR 'diagnostic screening' OR 'diagnostic screening programs' OR 'diagnostic sign' OR 'diagnostic tool' OR 'diagnostics' OR 'serology'/exp OR 'serologic reaction' OR 'serologic specificity' OR 'serologic survey' OR 'serologic technique' OR 'serologic test' OR 'serologic tests' OR 'serological characteristic' OR 'serological specificity' OR 'serological test' OR 'serology' OR 'fluorescent antibody technique'/exp OR 'antibody fluorescent technique' OR 'antibody, fluorescent' OR 'fluorescence antibody technique' OR 'fluorescence antibody test' OR 'fluorescence antiglobulin test' OR 'fluorescent antibody' OR 'fluorescent antibody darkfield method' OR 'fluorescent antibody identification' OR 'fluorescent antibody method' OR 'fluorescent antibody technique' OR 'fluorescent antibody test' OR 'fluorescent inhibition technique' OR 'enzyme linked immunosorbent assay'/exp OR 'elisa' OR 'enzyme labeled immunosorbent assay' OR 'enzyme linked immunoassay' OR 'enzyme linked immunosorbent assay' OR 'enzyme linked immunospecific assay' OR 'enzyme-linked immune assay' OR 'enzyme-linked immuno-assay' OR 'enzyme-linked immunosorbent assay' OR 'rapid test'/exp OR 'quick diagnostic test' OR 'rapid diagnosis test' OR 'rapid diagnostic laboratory test' OR 'rapid diagnostic test' OR 'rapid diagnostic test (rdt)' OR 'rapid diagnostic tests' OR 'rapid test' OR 'rapid testing' OR 'molecular diagnosis'/exp OR 'molecular diagnosis' OR 'molecular diagnostic' OR 'molecular diagnostic techniques' OR 'polymerase chain reaction'/exp OR 'pcr (polymerase chain reaction)' OR 'polymerase chain reaction' OR 'loop mediated isothermal amplification' OR 'LAMP (loop mediated isothermal amplification)' OR 'LAMP assay' OR 'LAMP assays' OR 'LAMP method' OR 'LAMP methods' OR 'LAMP procedure' OR 'LAMP procedures' OR 'LAMP reaction' OR 'LAMP reactions' OR 'LAMP test' OR 'LAMP testing' OR 'LAMP-based assay' OR 'LAMP-based method' OR 'loop mediated isothermal amplification assay' OR 'loop-mediated amplification' OR 'loop-mediated amplification (LAMP)' OR 'loop mediated isothermal amplification')
	**Outcome:**

	**#3** ('sensitivity and specificity'/exp OR 'sensitivity and specificity' OR 'specificity and sensitivity' OR 'sensitivity'/exp OR 'specificity'/exp OR 'diagnosis'/exp OR 'bacteriologic diagnosis' OR 'diagnosis' OR 'diagnostic screening' OR 'diagnostic screening programs' OR 'diagnostic sign' OR 'diagnostic tool' OR 'diagnostics' OR 'disease diagnosis' OR 'medical diagnosis' OR 'physical diagnosis' OR 'true positive result'/exp OR 'true negative result'/exp OR 'predictive value'/exp OR 'negative predictive value' OR 'positive predictive value' OR 'predictive value' OR 'predictive value of tests')	
	**Location:**<br>**#4** ('brazil'/exp OR 'brazil' OR 'federative republic of brazil' OR 'united states of brazil' OR 'brazilian' OR 'brazilians' OR 'brazilian'/exp)<br><br>**#5** [embase]/lim<br><br>#1 AND #2 AND #3 AND #4 AND #5	
Virtual Health Library (VHL)	**Population:**<br>**#1** (mh:(Esquistossomose mansoni)) OR (Esquistossomose mansoni) OR (mh:(Esquistosomiasis mansoni)) OR (Esquistosomiasis mansoni) OR (mh:(Schistosomiasis mansoni)) OR (Schistosomiasis mansoni) OR (Esquistossomose Mansônica) OR (Barriga D'água) OR (Esquistossomose Intestinal) OR (mh:(Esquistossomose)) OR (Esquistossomose) OR (mh:(Esquistosomiasis)) OR (Esquistosomiasis) OR (mh:(Schistosomiasis)) OR (Schistosomiasis) OR (Bilharzíase) OR (Infecção por Schistosoma) OR (Programa de Esquistossomose)<br>**Intervention:**<br>**#2** (mh:(Diagnóstico)) OR (Diagnóstico) OR (mh:(Diagnosis)) OR (Diagnosis) OR (Diagnose) OR (Detecção) OR (Exames e Diagnósticos) OR (mh:(Testes Sorológicos)) OR (Testes Sorológicos) OR (mh:(Pruebas Serológicas)) OR (Pruebas Serológicas) OR (mh:(Serologic Tests)) OR (Serologic Tests) OR (Testagem Sorológica) OR (Sorodiagnóstico) OR (Diagnóstico Sorológico) OR (mh:(Imunofluorescência)) OR (Imunofluorescência) OR (mh:(Técnica del Anticuerpo Fluorescente)) OR (Técnica del Anticuerpo Fluorescente) OR (mh:(Fluorescent Antibody Technique)) OR (Fluorescent Antibody Technique) OR (Testes de Anticorpos Fluorescentes Antinucleares) OR (Técnica de Imunofluorescência) OR (mh:(Ensaio de Imunoadsorção Enzimática)) OR (Ensaio de Imunoadsorção Enzimática) OR (mh:(Ensayo de Inmunoadsorción Enzimática)) OR (Ensayo de	206<br>(10/01/2024)

	Inmunoadsorción Enzimática) OR (mh:(Enzyme-Linked Immunosorbent Assay)) OR (Enzyme-Linked Immunosorbent Assay) OR (ELISA) OR (mh:(Testes de Diagnóstico Rápido)) OR (Testes de Diagnóstico Rápido) OR (mh:(Prueba de Diagnóstico Rápido)) OR (Prueba de Diagnóstico Rápido) OR (mh:(Rapid Diagnostic Tests)) OR (Rapid Diagnostic Tests) OR (Testes Rápidos de Diagnóstico) OR (Testes Rápidos) OR (Testes Diagnósticos Rápidos) OR (Testes Rápidos para Diagnóstico) OR (mh:(Técnicas de Diagnóstico Molecular)) OR (Técnicas de Diagnóstico Molecular) OR (mh:(Técnicas de Diagnóstico Molecular)) OR (Técnicas de Diagnóstico Molecular) OR (mh:(Molecular Diagnostic Techniques)) OR (Molecular Diagnostic Techniques) OR (Teste Molecular) OR (Teste Diagnóstico Molecular) OR (Diagnóstico Molecular) OR (Teste de Diagnóstico Molecular) OR (mh:(Reação em Cadeia da Polimerase)) OR (Reação em Cadeia da Polimerase) OR (mh:(Reacción en Cadena de la Polimerasa)) OR (Reacción en Cadena de la Polimerasa) OR (mh:(Polymerase Chain Reaction)) OR (Polymerase Chain Reaction) OR (PCR) OR (Reação da Polimerase em Cadeia) OR (Reação de Polimerase em Cadeia) OR (Reação em Cadeia de Polimerase) OR (PCR Reverso)<br>**Desfecho**<br>**#3** (Sensibilidade e Especificidade) OR (Sensitivity and Specificity) OR (Sensibilidad y Especificidad) OR (Sensibilidade) OR (Especificidade) OR (VerdadeirosNegativos) OR (VerdadeirosPositivos) OR (Valor Preditivo dos Testes) OR (Predictive Value of Tests) OR (Valor Predictivo de las Pruebas) OR (Valor Preditivo) OR (Valor PreditivoNegativo) OR (Valor PreditivoPositivo) OR (Valor Preditivo do Teste) OR (Valores Preditivos de Testes) OR (Reações Falso-Negativas) OR (Reações Falso-Positivas) OR (Diagnóstico) OR (Diagnosis) OR (Detecção) OR (Diagnose)<br>**Location:**<br>**#4** (mh:(Brasil)) OR (Brasil) OR (mh:(Brazil)) OR (Brazil)<br><br>**#5** (db:("LILACS" OR "SES-SP" OR "colecionaSUS" OR "campusvirtualsp_brasil" OR "BDENF" OR "IBECS" OR "PAHO" OR "PAHOIRIS" OR "HISA" OR "PREPRINT-SCIELO" OR "VETINDEX" OR "WPRIM"))<br><br>#1 AND #2 AND #3 AND #4 AND #5	
**Total (10/01/2024)**		1.745
