# Supplementary material for: Accuracy of diagnostic strategies for detecting Schistosoma mansoni infection in Brazil: A systematic review and meta-analysis
Source: Rev Soc Bras Med Trop. 2026 Aug 3;59:e0466-2025. doi: 10.1590/0037-8682-0466-2025 (PMC13432800; doi:10.1590/0037-8682-0466-2025)
Supplement: Supplementary File 2 (S2 File) [file 1678-9849-rsbmt-59-e0466-2025-md2.pdf]

S2 Table. **Immunological tests, ELISA and IFA, for the diagnosis of human schistosomiasis in Brazil**

Reference	Index test	Reference test	Reference test specification: n° of slides, n° of samples	Total number of cases	Total number of non-cases	Sensitivity (%)	Specificity (%)
Lopes et al. (2023)[60]	ELISA rSmME IgG	Kato-Katz	2 slides, 1 sample	53	65	81.1	46.1
Gonçalves et al. (2006)[45]	IF IgM SWAP	Kato-Katz or free sedimentation methods	12 slides, 3 samples	16	253	81.3	63.6
Gonçalves et al. (2006)[45]	ELISA SWAP IgG	Kato-Katz or free sedimentation methods	2 slides, 3 samples	16	253	87.5	54.9
Frota et al. (2011)[28]	ELISA SEA	Kato-Katz	8 slides, 4 samples	25	262	100	72.9
Carneiro et al. (2012)[15]	ELISA SWAP	Kato-Katz	3 slides, 1 sample	40	210	100	62.9
Oyeyemi et al. (2021)[58]	ELISA SCA	Kato-Katz and/or saline gradient	24 slides, 1 sample + 2 saline gradient	30	30	96.7	86.7
Oyeyemi et al. (2021)[58]	ELISA SEA	Kato-Katz e/ou saline gradient	24 slides, 1 sample + 2 saline gradient	30	30	90.0	86.7
Oyeyemi et al. (2021)[58]	ELISA SWAP	Kato-Katz e/ou saline gradient	24 slides, 1 sample + 2 saline gradient	30	30	90.0	96.7
Magalhães et al. (2023)[59]	ELISA SEA IgG1	Kato-Katz or HPJ or saline gradient or Helmintex®	18 slides, 1 sample	118	139	70	69.7
Magalhães et al. (2023)[59]	ELISA SEA IgG4	Kato-Katz or HPJ or saline gradient or Helmintex®	19 slides, 1 sample	118	139	78.4	65.6
Magalhães et al. (2023)[59]	ELISA SWAP IgG1	Kato-Katz or HPJ or saline gradient or Helmintex	22 slides, 1 sample	118	139	68.6	60

Magalhães et al. (2023)[59]	ELISA SWAP IgG4	Kato-Katz or HPJ or saline gradient or Helmintex	23 slides, 1 sample	118	139	73.5	61.1
Magalhães et al. (2023)[59]	ELISA SWAP-C IgG1	Kato-Katz or HPJ or saline gradient or Helmintex	20 slides, 1 sample	118	139	56.5	58.3
Magalhães et al. (2023)[59]	ELISA SWAP-C IgG4	Kato-Katz or HPJ or saline gradient or Helmintex	20 slides, 1 sample	118	139	72.8	60.5
Ramos et al. (2024)[19]	ELISA IgG (NovaLisa, Germany)	Helmintex	20 slides, 1 sample	85	130	87	28.4
Ramos et al. (2024)[19]	ELISA IgM (NovaLisa, Germany)	Helmintex	20 slides, 1 sample	63	137	55.5	43.8
