# Supplementary material for: Accuracy of diagnostic strategies for detecting Schistosoma mansoni infection in Brazil: A systematic review and meta-analysis
Source: Rev Soc Bras Med Trop. 2026 Aug 3;59:e0466-2025. doi: 10.1590/0037-8682-0466-2025 (PMC13432800; doi:10.1590/0037-8682-0466-2025)
Supplement: Supplementary File 3 (S3 File) [file 1678-9849-rsbmt-59-e0466-2025-md3.pdf]

S3 Table. POC-CCA for the diagnosis of human schistosomiasis in Brazil

Reference	Index test	Sample type used for the index test	Reference test	Reference test specification: number of slides, number of samples	Total number of cases	Total number of non-cases	Sensitivity (%)	Specificity (%)
Coelho et al. (2016)[49]	POC-CCA T+	Urine	Kato-Katz or saline gradient	24 slides, 1 sample (Kato-Katz) and 1g (saline gradient)	18	66	6.0	100
Coelho et al. (2016)[49]	POC-CCA T+	Lyophilized urine	Kato-Katz or saline gradient	25 slides, 1 sample (Kato-Katz) and 1g (saline gradient)	18	66	56.0	83.0
Siqueira et al. (2016)[50]	POC-CCA T+	Urine	Kato-Katz or saline gradient	24 slides, 1 sample + 2 liquots, 1 sample	34	107	73.5	93.5
Siqueira et al. (2016) [50]	POC-CCA T+	Urine	Kato-Katz	24 slides, 1 sample	27	114	77.8	90.4
Siqueira et al. (2016) [50]	POC-CCA T+	Urine	Saline gradient	2 alíquotas, 1 sample	24	117	83.3	89.7
Siqueira et al. (2016) [50]	POC-CCA T+	Urine	Kato-Katz	2 slides, 1 sample	15	126	86.7	84.9
Ferreira et al. (2017)[51]	POC-CCA T+	Urine	Kato-Katz	13 slides, 3 samples	26	274	57.7	75.5
Ferreira et al. (2017)[51]	POC-CCA T+	Urine	Kato-Katz	1 slide, 1 sample	14	286	64.3	74.5
Ferreira et al. (2017)[51]	POC-CCA T+	Urine	Kato-Katz	4 slides, 2 samples	21	279	52.4	75.3

Ferreira et al. (2017)[51]	POC-CCA T+	Urine	Kato-Katz	6 slides, 3 samples	26	274	55.5	75.7
Ferreira et al. (2017)[51]	POC-CCA T+	Urine	Kato-Katz	2 slides, 1 sample	18	282	61.1	74.8
Lindholz et al. (2018)[16]	POC-CCA T-	Urine	Kato-Katz	2 slides, 1 sample	55	406	72.7	63.8
Lindholz et al. (2018)[16]	POC-CCA T-	Urine	Helmintex	1 sample	187	274	57.2	70.8
Lindholz et al. (2018)[16]	POC-CCA T+	Urine	Kato-Katz	2 slides, 1 sample	55	406	85.5	30.3
Lindholz et al. (2018)[16]	POC-CCA T+	Urine	Helmintex	1 sample	187	274	81.8	35.4
Oliveira et al. (2018)[17]	POC-CCA T-	Urine	Kato-Katz, saline gradient and Helmintex	18 slides (Kato-Katz), 500mg (saline gradient), 30g (Helmintex)	112	116	26.8	98.3
Oliveira et al. (2018)[17]	POC-CCA T+	Urine	Kato-Katz or saline gradient or helmintex	18 slides (Kato-Katz), 500mg (saline gradient), 30g (Helmintex)	112	116	65.2	69.8
Queiroz et al. (2018)[53]	POC-CCA	lyophilized urine	Kato-Katz and Gradiente Salino	24 slides, 1 sample + 2 aliquots, 1 sample	32	52	56	83
Queiroz et al. (2018)[53]	POC-CCA	Urine	Kato-Katz and saline gradient	24 slides, 1 sample + 2 aliquots, 1 sample	32	52	6.0	100
Grenfell et al. (2019)[9]	POC-CCA T-	Urine	Kato-Katz and saline gradient	24 slides (Kato-Katz) + 2	21	27	23.8	81.5

				analyses of 500mg				
Grenfell et al. (2019)[9]	POC-CCA	Concentrated urine	Kato-Katz and saline gradient	24 slides (Kato-Katz) + 2 analyses of 500mg	21	27	76.2	85.2
Grenfell et al. (2019)[9]	POC-CCA T+	Urine	Kato-Katz or saline gradient	24 slides (Kato-Katz) + 2 analyses of 500mg	21	27	81.0	59.3
Grenfell et al. (2019)[9]	POC-ECO T+	Urine	Kato-Katz and saline gradient	24 slides (Kato-Katz) + 2 analyses of 500mg	21	27	33.3	70.4
Grenfell et al. (2019)[9]	POC-ECO T-	Urine	Kato-Katz and saline gradient	24 slides (Kato-Katz) + 2 analyses of 500mg	21	27	14.3	78.0
Souza et al. (2019)[55]	POC-CCA T-	Urine	Kato-Katz	16 slides, 3 samples	35	338	17.1	97.0
Souza et al. (2019)[55]	POC-CCA T+	Urine	Kato-Katz	1 slide, 1 sample	9	363	55.6	76.9
Souza et al. (2019)[55]	POC-CCA T+	Urine	Kato-Katz	6 slides, 3 samples	24	348	58.3	78.4
Souza et al. (2019)[55]	POC-CCA T+	Urine	Kato-Katz	16 slides, 3 samples	35	337	65.7	80.4
Sousa et al. (2020)[55]	POC-CCA T-	Urine	Kato-Katz and Helmintex	16 slides (12 slides first sample + 2 slides second sample +	63	154	36.5	90.9

				2 slides third sample) + 30g of feces for Helmintex				
Sousa et al. (2020)[55]	POC-CCA T+	Urine	Kato-Katz and Helmintex	16 slides (12 slides first samples + 2 slides second samples + 2 slides third sample) + 30g of feces for Helmintex	63	154	61.9	74.0
Sousa et al. (2020)[55]	POC-CCA T+	Urine	Helmintex	1 sample	53	164	56.6	70.1
Sousa et al. (2020)[55]	POC-CCA T+	Urine	Kato-Katz	1 slide, 1 sample	12	205	83.3	66.3
Sousa et al. (2020)[55]	POC-CCA T+	Urine	Kato-Katz	2 slides, 1 sample	17	200	88.2	68.0
Sousa et al. (2020)[55]	POC-CCA T+	Urine	Kato-Katz	16 slides (12 slides first sample + 2 slides second samples + 2 slides third sample)	31	186	80.6	71.0
Bezerra et al. (2021)[20]	POC-CCA T-	Urine	Kato-Katz	6 slides, 3 samples	62	65	74.2	83.1
Bezerra et al. (2021)[20]	POC-CCA T+	Urine	Kato-Katz	6 slides, 3 samples	62	65	85.5	52.3

Pieri et al. (2023)[61]	POC-ECO T-	Urine	Helmintex®	1 sample	424	1471	57.8	80.6
Pieri et al. (2023)[61]	POC-ECO T+	Urine	Helmintex®	1 sample	424	1471	80.2	55.0
Ramos et al. (2024)[19]	ICT IgG/IgM	Serum	Helmintex®	1 sample	70	117	95.7	18.8
