# Supplementary material for: Accuracy of diagnostic strategies for detecting Schistosoma mansoni infection in Brazil: A systematic review and meta-analysis
Source: Rev Soc Bras Med Trop. 2026 Aug 3;59:e0466-2025. doi: 10.1590/0037-8682-0466-2025 (PMC13432800; doi:10.1590/0037-8682-0466-2025)
Supplement: Supplementary File 4 (S4 File) [file 1678-9849-rsbmt-59-e0466-2025-md4.pdf]

S4 Table. Molecular tests for the diagnosis of human schistosomiasis in Brazil

Reference	Index test	Reference test	Reference test specification: n° of slides, n° of samples	Total number of cases	Total number of non-cases	Sensitivity (%)	Specificity (%)
Pontes et al. (2003)[44]	PCR	Kato-Katz	2 slides, 3 samples	60	134	96.7	88.1
Gomes et al. (2009)[46]	PCR	Kato-Katz	6 slides, 1 sample	28	39	92.6	62.0
Gomes et al. (2010)[47]	PCR-ELISA	Kato-Katz	12 slides, 1 sample	38	168	97.4	85.1
Gomes et al. (2010)[47]	PCR-ELISA	Kato-Katz	2 slides, 1 sample	27	179	96.3	79.9
Oliveira et al. (2010)[29]	PCR	Kato-Katz	3 samples	34	46	73.5	100
Oliveira et al. (2010)[29]	PCR SmF/SmR	Kato-Katz	3 samples	34	46	82.4	100
Siqueira et al. (2021)[23]	PCR-ELISA	Kato-Katz	12 samples	29	172	55.2	88.3
Siqueira et al. (2021)[23]	PCR-ELISA	Kato-Katz	1 slide	16	185	68.8	86.5
Carvalho et al. (2012)[21]	PCR	Kato-Katz	2 slides, 1 sample	16	203	93.8	70.0
Enk et al. (2012)[43]	PCR	Kato-Katz, saline gradient and miracidia hatching technique	Kato-Katz: 18 slides, 4 samples. SG: 500mg, 1 sample. MHT: 1g, 1 sample	69	125	100	91,2
Gandasegui et al. (2018)[25]	LAMP SmMIT	Kato-Katz	2 slides, 1 sample	13	149	93.0	80.1
Senra et al. (2018)[54]	PCR-ELISA (commercial platform)	Kato-Katz	12 slides	38	168	97.4	85.1
Senra et al. (2018) [54]	PCR-ELISA (commercial platform)	Kato-Katz	12 slides	38	168	97.4	91.1
Magalhães et al. (2020)[22]	qPCR	Kato-Katz or saline gradient or Helmintex	18 slides, 3 samples ou 1 saline gradient ou 1 Helmintex	104	111	91.4	80.2

Siqueira et al. (2021)[23]	qPCR (Estreito de Miralta)	Kato-Katz	2 slides, 1 sample	15	127	80.0	89.0
Siqueira et al. (2021)[23]	qPCR (Estreito de Miralta)	Kato-Katz	24 slides, 1 sample	28	114	64.3	92.9
Siqueira et al. (2021)[23]	qPCR (Estreito de Miralta)	Saline gradient	1000mg, 1 sample	26	116	69.2	93.1
Siqueira et al. (2021)[23]	qPCR (Estreito de Miralta)	Kato-Katz or saline gradient	24 slides, 1 sample (Kato-Katz, 1000mg, 1 sample (saline gradient)	35	107	57.1	94.4
Siqueira et al. (2021)[23]	qPCR (Tabuas)	Kato-Katz	2 slides, 1 sample	23	125	95.7	81.6
Siqueira et al. (2021)[23]	qPCR	qPCR	24 slides, 1 sample	31	117	96.8	87.2
Siqueira et al. (2021)[23]	qPCR (Tabuas)	Saline gradient	1000mg, 1 sample	43	105	81.4	90.5
Siqueira et al. (2021)[23]	qPCR (Tabuas)	Kato-Katz or saline gradient	24 slides, 1 sample (Kato-Katz, 1000mg, 1 samples (saline gradient)	46	102	82.6	93.1
Gomes et al. (2022)[26]	LAMP SmITS1	Kato-Katz	Kato-Katz	144	178	11.8	93.3
