# Supplementary material for: Accuracy of diagnostic strategies for detecting Schistosoma mansoni infection in Brazil: A systematic review and meta-analysis
Source: Rev Soc Bras Med Trop. 2026 Aug 3;59:e0466-2025. doi: 10.1590/0037-8682-0466-2025 (PMC13432800; doi:10.1590/0037-8682-0466-2025)

**S5 Table. Parasitological tests for the diagnosis of human schistosomiasis in Brazil**

| Reference | Index test | Reference test | Reference test specification: number of slides, number of samples | Total number of cases | Total number of non-cases | Sensitivity (%) | Specificity (%) |
|---|---|---|---|---|---|---|---|
| Nacife et al. (2018)[52] | TF-Test | Kato-Katz | 1 slide de 1 sample | 249 | 296 | 59 | 88.5 |
| Siqueira et al. (2011)[56] | TF-Test | Kato-Katz | 3 slides, 3 samples | 37 | 164 | 16.2 | 84.2 |
| Siqueira et al. (2011)[56] | TF-Test | Kato-Katz | 6 slides, 3 samples | 44 | 157 | 29.6 | 87.9 |
| Lindholz et al. (2018)[16] | Helmintex | Kato-Katz | 3 slides, 1 sample | 55 | 406 | 98.2 | 67.2 |
| Pieri et al., (2023)[61] | Kato-Katz | Helmintex | 1 sample | 424 | 1471 | 41.3 | 97.4 |

Figure. Pooled accuracy estimates of the TF-Test for the diagnosis of *Schistosoma mansoni* infection in Brazil

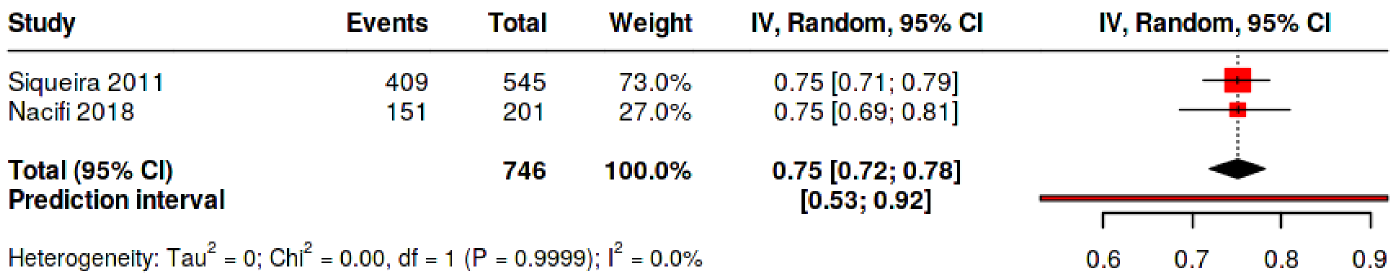

Supplement: Supplementary File 5 (S5 File) [file 1678-9849-rsbmt-59-e0466-2025-md5.pdf]
