# Supplementary material for: Accuracy of diagnostic strategies for detecting Schistosoma mansoni infection in Brazil: A systematic review and meta-analysis
Source: Rev Soc Bras Med Trop. 2026 Aug 3;59:e0466-2025. doi: 10.1590/0037-8682-0466-2025 (PMC13432800; doi:10.1590/0037-8682-0466-2025)
Supplement: Supplementary File 6 (S6 File) [file 1678-9849-rsbmt-59-e0466-2025-md6.pdf]

S6 File. Pooled estimates of sensitivity and specificity for the tests evaluated for the diagnosis of schistosomiasis in Brazil

1. **Pooled estimates of sensitivity and specificity of SEA-ELISA (a,b) and SWAP-ELISA (c,d)**

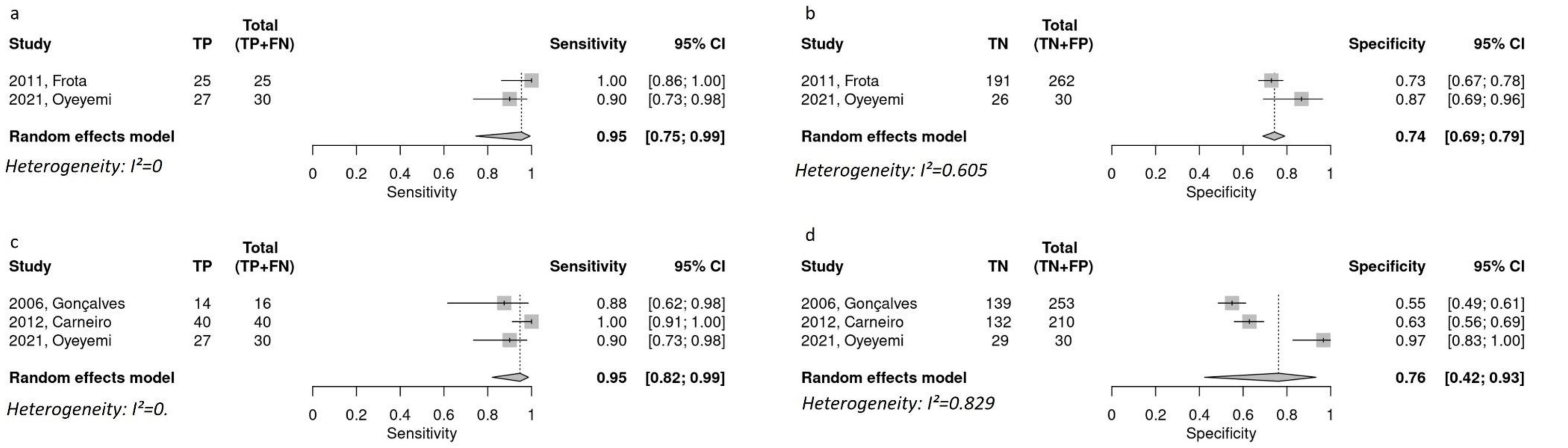

Legend. SEA-ELISA: sensitivity (a) and specificity (b); SWAP-ELISA: sensitivity (c) and specificity (d).

2. Pooled estimates of sensitivity and specificity for POC-CCA considering trait as positive (a,b) and as negative (c,d)

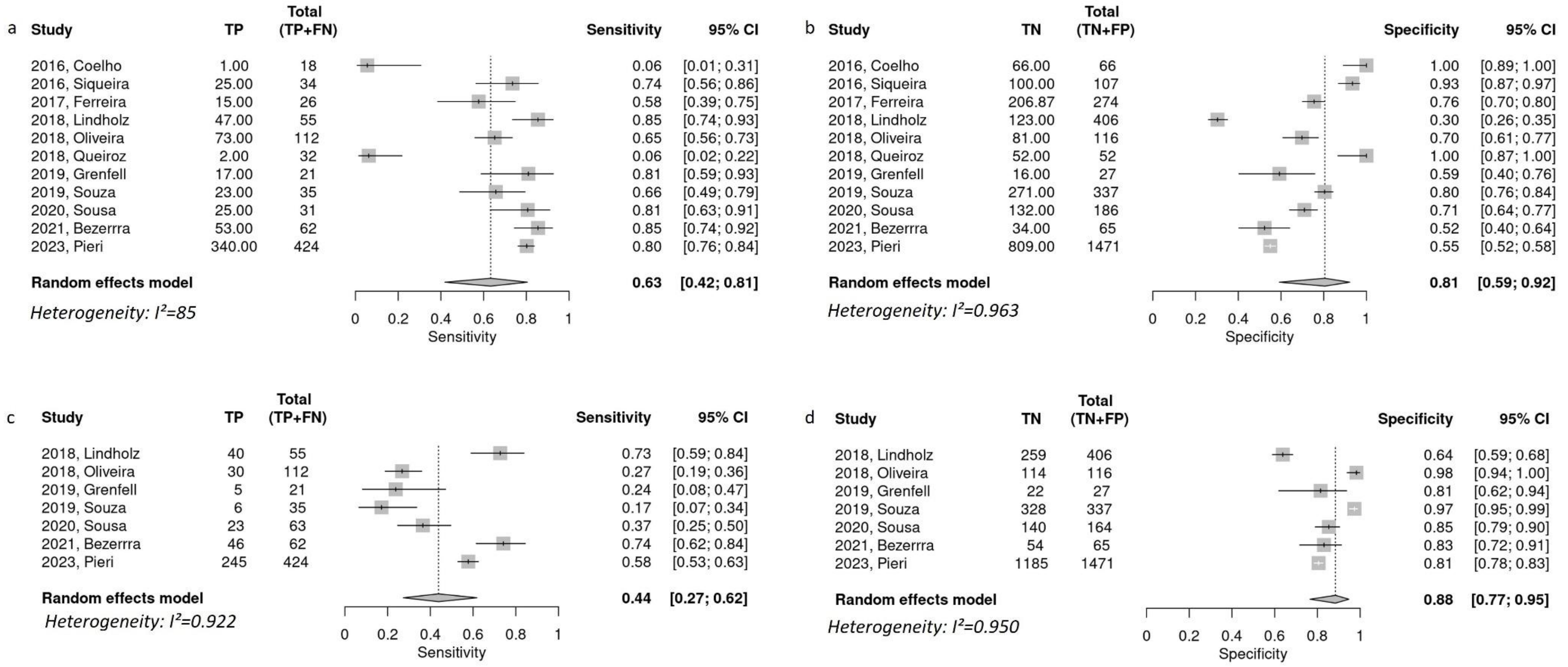

Legend. POC-CCA (positive trait): sensitivity (a) and specificity (b); POC-CCA (negative trait): sensitivity (c) and specificity (d).

3. Pooled estimates of sensitivity and specificity of conventional PCR (a, b), PCR-ELISA (c, d), real-time PCR (e, f) and LAMP (g, h) for the diagnosis of schistosomiasis in Brazil.

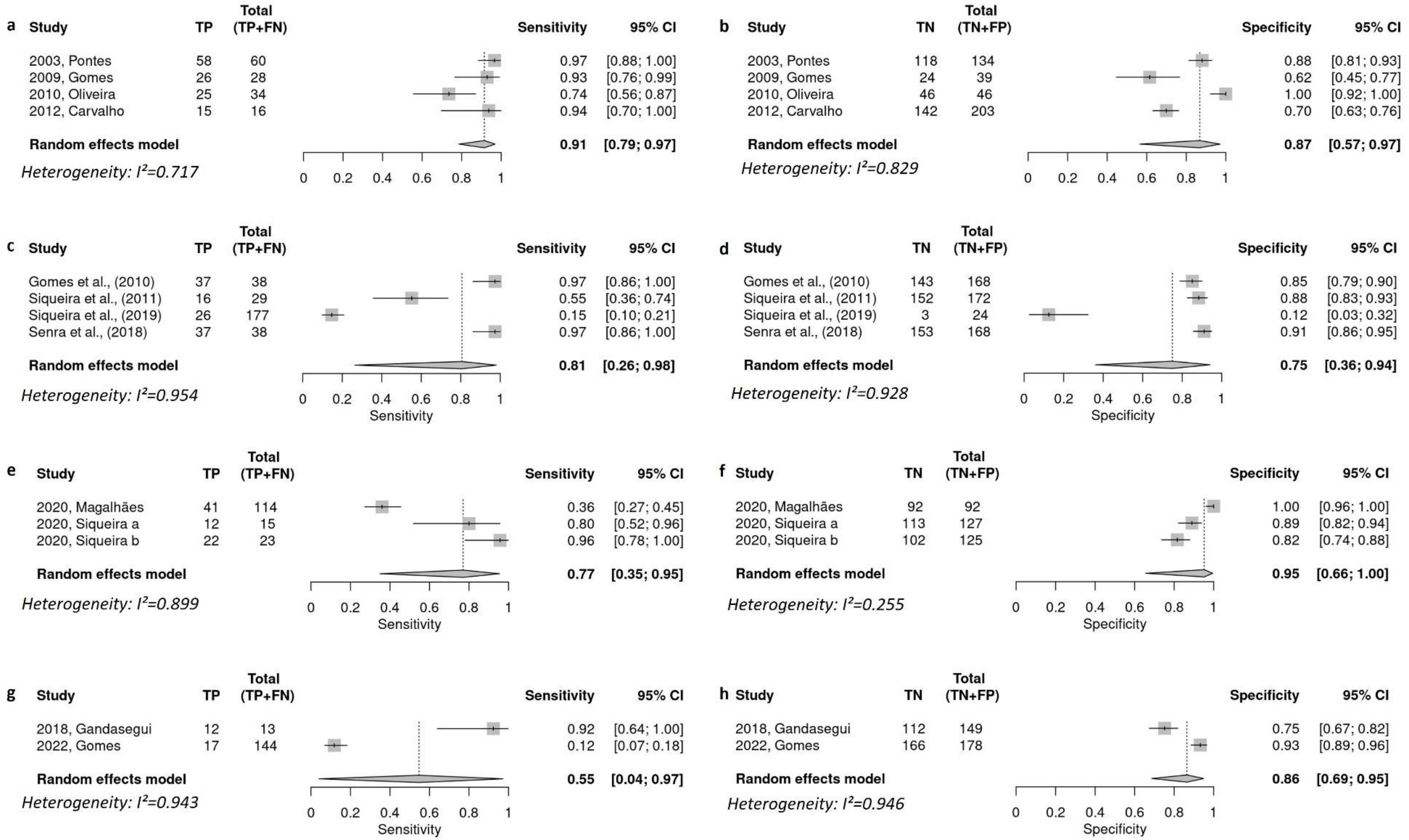

Legenda.

Legend. Conventional PCR: sensitivity (a) and specificity (b); PCR-ELISA: sensitivity (c) and specificity (d); Real-time PCR: sensitivity (e) and specificity (f); LAMP: sensitivity (g) and specificity (h)

4. Pooled estimates of sensitivity and specificity of the TF-Test (a,b) for the diagnosis of schistosomiasis in Brazil

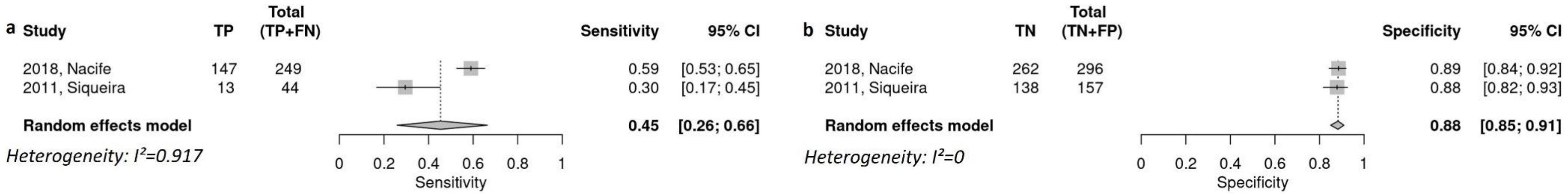

Legend. TF-Test: sensitivity (c) and specificity
