# Supplementary material for: Accuracy of diagnostic strategies for detecting Schistosoma mansoni infection in Brazil: A systematic review and meta-analysis
Source: Rev Soc Bras Med Trop. 2026 Aug 3;59:e0466-2025. doi: 10.1590/0037-8682-0466-2025 (PMC13432800; doi:10.1590/0037-8682-0466-2025)

S7 File. Risk of bias assessment using the QUADAS-2 tool for diagnostic accuracy studies of human *Schistosoma mansoni* infection

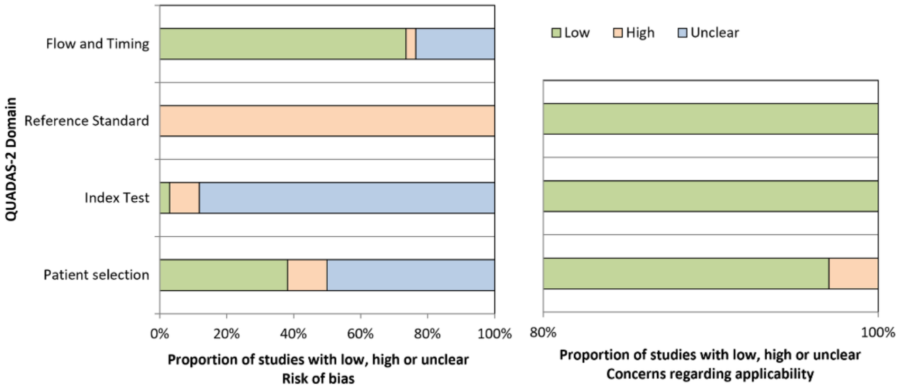

Supplement: Supplementary File 7 (S7 File) [file 1678-9849-rsbmt-59-e0466-2025-md7.pdf]
